# Cholinergic and Glutamatergic Axons Differentially Require Glial Support in the Drosophila PNS

**DOI:** 10.1002/glia.70011

**Published:** 2025-03-17

**Authors:** Steffen Kautzmann, Simone Rey, Amber Krebs, Christian Klämbt

**Affiliations:** ^1^ Institut für Neuro‐ Und Verhaltensbiologie Universität Münster Münster Germany

**Keywords:** APEX2, axon, axonal degeneration, Drosophila, neuron glia interaction

## Abstract

In vertebrates, there is a differential interaction between peripheral axons and their associated glial cells. While large‐caliber axons are covered by a myelin sheath, small‐diameter axons are simply wrapped in Remak fibers. In peripheral nerves of Drosophila larvae, axons are covered by wrapping glial cell processes similar to vertebrate Remak fibers. Whether differences in axonal diameter influence the interaction with glial processes in Drosophila has not yet been analyzed. Likewise, it is not understood whether the modality of the neuron affects the interaction with the wrapping glia. To start to decipher the mechanisms underlying glial wrapping, we employed APEX2 labeling in larval filet preparations. This allowed us to follow individual axons of defined segmental nerves at ultrastructural resolution in the presence or absence of wrapping glia. Using these tools, we first demonstrate that motor axons are larger compared to sensory axons. Sensory axons fasciculate in larger groups than motor axons, suggesting that they do not require direct contact with wrapping glia. However, unlike motor axons, sensory axons show length‐dependent degeneration upon ablation of wrapping glia. These data suggest that Drosophila may help to understand peripheral neuropathies caused by defects in Schwann cell function, in which a similar degeneration of sensory axons is observed.

## Main Points

- In the Drosophila PNS, motor‐ and sensory axons differentially interact with wrapping glia processes.
- Sensory axons are more weakly associated with wrapping glia and show a length dependent degeneration upon loss of wrapping glia.

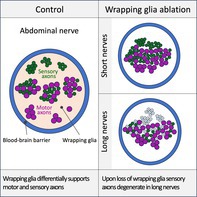

## 1 Introduction

Neurons transmit information via often very long axons. In the peripheral nervous system (PNS) of vertebrates, the length of an axon can reach several meters, and even in the comparably small PNS of Drosophila larvae, the length of the axons can be almost 1000‐fold larger than the diameter of the neuronal cell body. In order to cope with the metabolic problems that are inevitably consequences of long and thin cell processes, neighboring glial cells evolved that are able to provide the necessary metabolic and structural support (Fünfschilling et al. 2012; Lee et al. 2012; Nave and Werner 2021; Rey et al. 2021; Volkenhoff et al. 2015).

The vertebrate glial cells that accompany axons in the PNS are called Schwann cells (Jessen et al. 2015; Kidd et al. 2013). The differential interaction of axons with neighboring Schwann cells is dictated by the radial diameter of the axons. Large axons become myelinated, while small‐caliber axons are found in Remak fibers (Boullerne 2016; Feltri et al. 2016; Michailov et al. 2004; Nave and Werner 2014, 2021; Taveggia et al. 2008). A contribution of neuronal modality in neuron–glia interaction has not yet been described.

Quite similarly, in the Drosophila larval PNS, wrapping glial cells interact with the axons that are located in common segmentally arranged nerves. Every abdominal nerve harbors about 80 axons and only three wrapping glial cells that are consecutively arranged along the entire length of the nerve (Kottmeier et al. 2020; Matzat et al. 2015; von Hilchen et al. 2013). Following retrograde DiI labeling, 34 or 31–32 motor neurons were identified in abdominal neuromeres of late embryos (Landgraf et al. 1997; Sink and Whitington 1991). Further single neuroblast labeling experiments identified 32 motor neurons in abdominal hemineuromeres (Schmid et al. 1999). These embryonic motor neurons innervate the 30 muscles localized in each abdominal hemisegment (Matthias Landgraf and Thor 2006). All Drosophila motor neurons are glutamatergic, of which some additionally release octopamine/tyramine or other neuropeptides (Sherer et al. 2020). In addition, 43 cholinergic sensory neurons have been identified in the abdominal body wall that project their axons towards the ventral nerve cord (Ghysen and Dambly‐Chaudière 1993). Moreover, non‐cholinergic and non‐glutamatergic peptidergic neurons exist, such as a single leucokinergic neuron that is present only in abdominal hemineuromeres A1–A7 and projects a single axon to the periphery (de Haro et al. 2010). Similarly, one pair of Crustacean CardioActive Peptide (CCAP) expressing neurons is found in a subset of abdominal hemineuromeres (A1–A4) that project their axons towards the periphery (Karsai et al. 2013; Santos et al. 2007). In addition, several Gal4‐driver lines are reported that label single or small subsets of central nervous system (CNS) neurons that project their axons only in a subset of abdominal nerves (Karsai et al. 2013; Qian et al. 2018; Santos et al. 2007). Thus, the number of axons is not constant across abdominal nerves but varies between 76 and 80 axons.

During embryonic development, motor axons grow out first. Along the motor axons, CNS‐derived glial cells migrate into the forming segmental nerves utilizing the guidance molecule Netrin (Sepp et al. 2000; von Hilchen et al. 2010). Notch as well as the Ig‐domain containing adhesion protein Fasciclin2 are subsequently used to adjust their final positioning (Edenfeld et al. 2007; Neuert et al. 2020; Sepp et al. 2000; Silies and Klämbt 2010; von Hilchen et al. 2010). Sensory axons then follow the path preestablished by motor axons towards the CNS. All axons together with their wrapping glial cells are engulfed by seven glial cells, four subperineurial and three perineurial glial cells that form the blood–brain barrier along each of the abdominal nerves (Matzat et al. 2015; Stork et al. 2008; von Hilchen et al. 2008, 2013).

The embryonic wrapping glial cells are simple elongated cells that do not yet engulf axons (Stork et al. 2008). During larval development, the morphological complexity of the wrapping glial cells gradually increases. First, the wrapping glial cells grow thin processes that grow along the entire length of the nerve, and then start enwrapping smaller axon fascicles or individual axons (Stork et al. 2008). Similar to vertebrates, the differentiation of the axon wrapping glial cells depends on receptor tyrosine kinase signaling. At the end of larval development, a Remak‐fiber‐like wrapping mode can be observed (Matzat et al. 2015; Stork et al. 2008).

During pupal stages, the larval nerves undergo extensive remodeling (Subramanian et al. 2017). In addition to motor axons that innervate the adult musculature, many sensory neurons are formed in the adult fly, resulting in about 760 axons in each of the leg nerves (Rey et al. 2023). Interestingly, adult wrapping glia engulf axons in a more sophisticated manner as compared to larval stages, resulting in myelin‐like structures that form close to the axon initial segments in the leg nerves (Rey et al. 2023). Here, glial cells form numerous thin glial processes that first establish a lacunar system around the axon initial segments of only large‐caliber motor axons and later collapse to form myelin‐like structures along these axons (Rey et al. 2023). How the axon‐ensheathing glia recognize the relevant axons is currently unknown.

Here we addressed whether axons belonging to different neuronal modalities are spatially organized or whether axons are running with random neighborhood relationships within the segmental nerves. Our data favor the existence of an axon map and demonstrate that motor axons show a stronger association with glial cells. Surprisingly, we noted that although motor axons appear to establish more intensive glial contacts than sensory axons, they do not depend on the presence of glial processes. In stark contrast, sensory axons are crucially dependent on the presence of wrapping glia, and in the absence of these glial cells, they show a length‐dependent degeneration. This suggests that studies of length‐dependent degeneration of Drosophila sensory axons upon loss of wrapping glia may contribute to a better mechanistic understanding of peripheral neuropathies in vertebrates, where loss of axonal ensheathment leads to damage of sensory axons in the PNS.

## 2 Results

### 2.1 Labeling of Individual Axons Within Nerves

**FIGURE 1:**
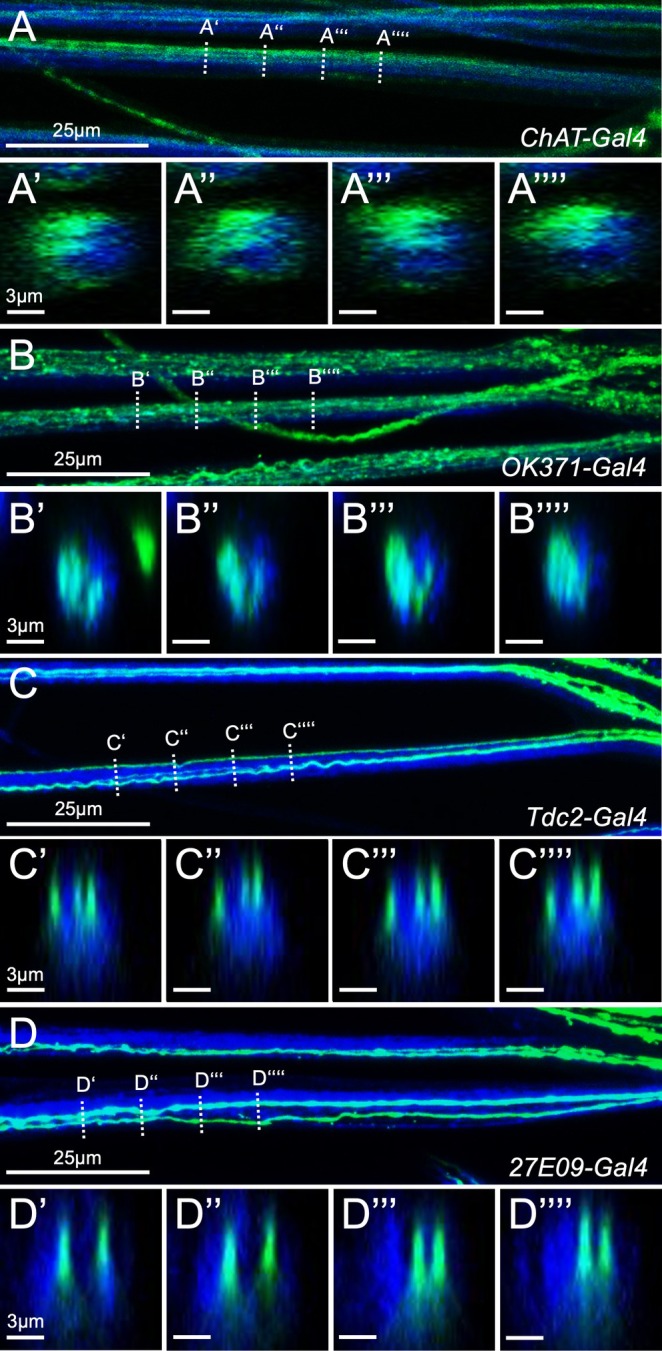
Confocal analysis of abdominal nerves in third instar larval filet preparations. Maximum intensity projections of larval filet expressing CD8::GFP directed by the Gal4 elements indicated (green). Neuronal membranes are shown in blue (anti‐HRP staining). (A) Labeling of all cholinergic neurons. The dashed lines indicate the positions of the orthogonal sections shown in (A′–A″). (B) Labeling of all glutamatergic neurons. The dashed lines indicate the positions of the orthogonal sections shown in (B′–'‐B″). (C) Labeling of all octopaminergic/tyraminergic neurons. The dashed lines indicate the positions of the orthogonal sections shown in (C′–C″). (D) Labeling of the two *27E09‐Gal4* positive motor neurons. The dashed lines indicate the positions of the orthogonal sections shown in (D′–D″). Note, that the spatial relationship of the different axons appears conserved.

To establish a map of axon identities within the nerve, we selected Gal4 driver lines that are active in specific neuronal subsets (Li et al. 2014) (see Section 4). To target all sensory neurons, we employed a driver harboring enhancer sequences of the *Choline acetyltransferase* (*Chat*) gene [*ChAT‐Gal4*] that is active in all cholinergic neurons (Salvaterra and Kitamoto 2001). To visualize all motor axons, we used a Gal4 element inserted in the *vesicular Glutamate transporter* (*vGlut*) gene that is active in all glutamatergic neurons [*OK371‐Gal4*] (Mahr and Aberle 2006). When these Gal4 driver strains were used to activate expression of CD8::GFP, the axonal trajectories can be easily identified in larval nerves (Figure 1A,B), and a distinct localization of either motor as well as sensory axons can be observed in orthogonal views of confocal images (Figure 1A'‐ A'''',B'‐ B'''').

In addition, we used *Tdc2‐Gal4* to target three octopaminergic/tyraminergic neurons and *27E09‐Gal4* to label only two specific glutamatergic motor neurons per hemineuromer. When these Gal4 driver strains were used to activate the expression of CD8::GFP, the axonal trajectories were easy to identify in larval nerves (Figure 1C,D). In consecutive orthogonal views of such nerves, it is apparent that specific axons are generally running with a similar spatial arrangement (Figure 1C′–C‴,D′–D‴). This suggests an ordered localization of axons within the nerve.

### 2.2 Electron Microscopic Imaging

**FIGURE 2:**
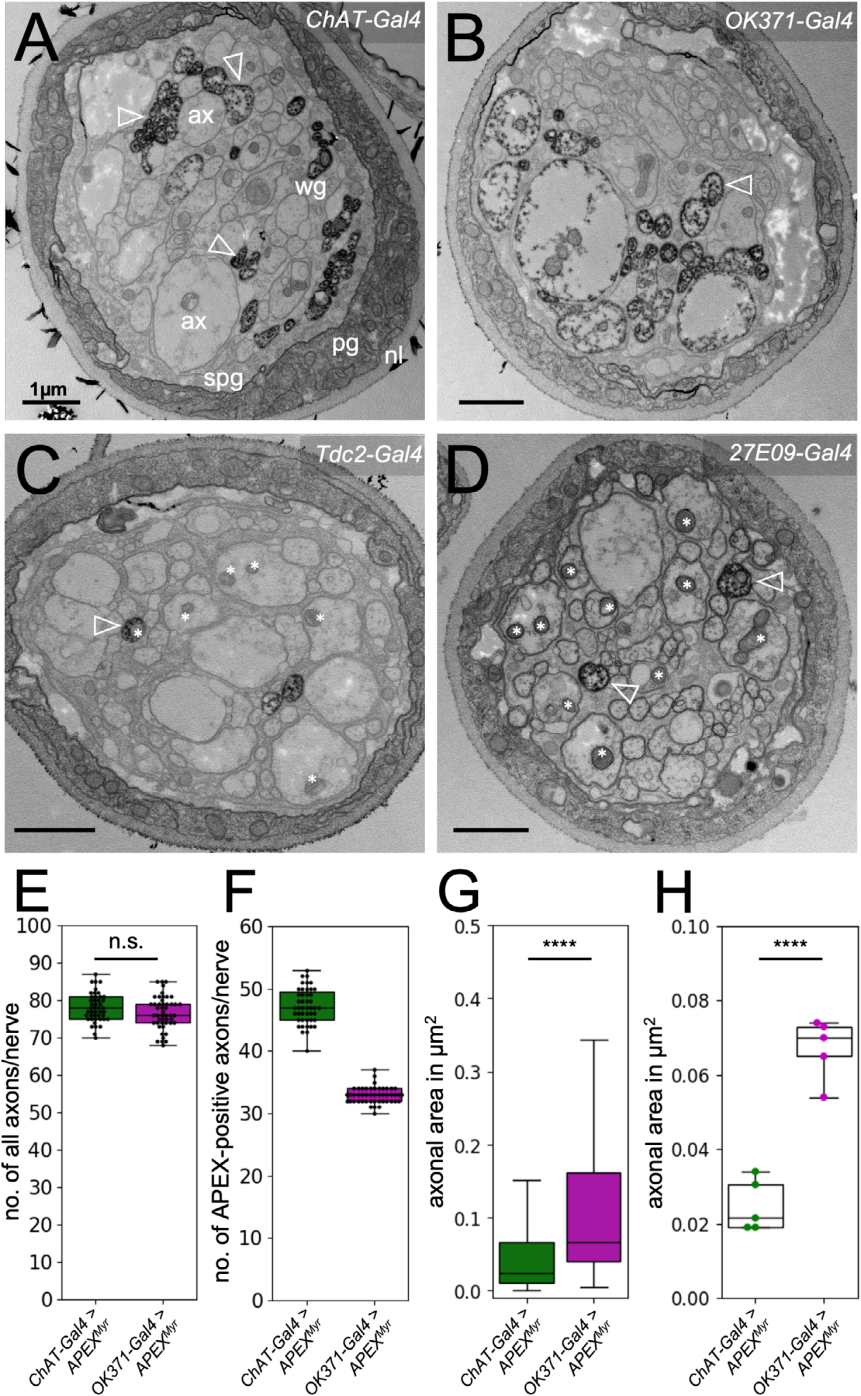
Electron microscopic images of cross sections of peripheral nerves from third instar larvae expressing APEX2^myr^ using the Gal4‐drivers indicated. APEX2^myr^ activity was used to generate an osmiophilic DAB precipitate, which can be recognized by dark labeling (white arrowheads). (A) *ChAT‐Gal4* activity allows the labeling of all sensory axons clustering to the outer surface of one half of the nerve. (B) *OK371‐Gal4* activity results in the labeling of all motor axons, which cluster into one half of the nerve. (C) *Tdc2‐Gal4* activity allows the labeling of three axons per nerve. (D) *27E09‐Gal4* activity allows the labeling of two motor axons. Note the presence of mitochondria in most large diameter axon (white asterisks). Scale bar is 1 μm in all images. ax, axon; nl, neural lamella; pg, perineurial glia; spg, subperineurial glia; wg, wrapping glia. (E) Quantification of the total number of axons per nerve of animals expressing APEX2^myr^ either in cholinergic (green shading) or in glutamatergic neurons (magenta shading). This color‐coding scheme is the same for all figures. Bottom and top of boxplots represent 25% and 75% percentile, respectively. Whiskers represent lower and upper quartile. The total number of axons does not significantly differ between both genotypes. (F) Quantification of the number of APEX‐positive axons and (G) their axonal area from both genotypes each. Around 47 cholinergic and 33 glutamatergic axons can be found in peripheral nerves. (H) Quantification of the median axonal areas determined in five individual animals. Glutamatergic axons exhibit a larger diameter compared to cholinergic axons. For details on the statistical analysis see Table 1.

To obtain a higher resolution level, we turned to the electron microscope. To determine the spatial arrangement of axons in a given nerve, we generated filet preparations, as previously described, that also allowed the identification of nerve identities based on the localization of nerves within the section (Matzat et al. 2015). To allow visualization of specific axons in electron micrographs, peroxidases such as horseradish peroxidase (HRP) or variants of the soybean ascorbate peroxidase (APEX) can be expressed (Becker et al. 2023; Dubois et al. 2001; Lam et al. 2015; Matzat et al. 2015). We employed a myristoylated APEX2 (APEX2^myr^) peroxidase to generate an insoluble and osmiophilic diaminobenzidine (DAB) precipitate, which can be easily localized in the electron microscope after osmium treatment (Rey et al. 2023; Tsang et al. 2018). Expression of APEX2^myr^ in neurons does not interfere with viability. APEX2^myr^ expressing flies eclose in normal numbers and show no discernable phenotypes. When we expressed APEX2^myr^ in subsets of neurons using the driver lines mentioned above, the corresponding axons were reliably detectable after sectioning by their high contrast (Figure 2A–D). Similarly, as noted in the confocal microscope, motor axons and sensory axons appear to segregate in distinct domains within larval abdominal nerves using electron microscopic analyses (Figure 2A,B). Sensory axons are generally very small in diameter and fasciculate in groups, often at the outer surface of one half of the nerve (Figure 2A,G). Motor axons are generally larger and occupy clearly distinct territories in the nerve, comparable to what we have detected using the confocal microscope (Figure 2B,G). Likewise, when we labeled only smaller axonal subsets, we noted their stereotyped positioning previously detected in confocal images (Figures 1C,D and 2C,D).

### 2.3 Differential Properties of Cholinergic and Glutamatergic Axons

To determine the properties of cholinergic and glutamatergic axons in greater detail, we sectioned correspondingly stained larval filets at a 150 μm distance from the tip of the ventral nerve cord. This allows us to faithfully section the majority of abdominal nerves A2–A8. When we labeled all cholinergic sensory neurons using flies of the genotype [*ChAT‐Gal4, UAS‐APEX2* ^*myr*^], on average we detected 47 cholinergic axons out of 78 axons per nerve. The median cross‐sectional area of all cholinergic axons is 0.024 μm^2^, corresponding to a radius of 0.09 μm (Figure 2E–G; green shading indicates cholinergic neurons throughout the manuscript; see Table 1 for statistical analysis). We did not observe large differences in the median axonal areas of individual animals (Figure 2H, green dots).

**TABLE 1:** Statistics to Figure 2.

	(E) No. of all axons/nerve		(F) No. of APEX‐positive axons/nerve	
	(*ChAT>APEX2*)	(*OK371 > APEX2*)	(*ChAT>APEX2*)	(*OK371 > APEX2*)
Animals	5	5	5	5
Number of nerves	47	50	47	50
Median number of axons	78	76	47	33
Shapiro	0.2	0.32	0.018	0.0098
*p*‐value	0.07 (*t* test)		2.46 × 10^−17^ (MW test)	

	(G) Axonal area (pooled)		(H) Axonal area (individual animals)	
	(*ChAT>APEX2*)	(*OK371>APEX2*)	(*ChAT>APEX2*)	(*OK371>APEX2*)
Animals	5	5	5	5
Number of nerves	47	50	47	50
Median axonal area	0.024	0.067	0.0215	0.07
Axons	2217	1645	—	—
Shapiro	0	0	0.14	0.26
*p*‐value	3.98 × 10^−20^ (MW test)		2.14 × 10^−05^(*t* test)	

*Note*: The numbers of analyzed animals and nerves is indicated. The Shapiro test was used to test for normal distribution of the data. Upon normal distribution a *t* test was used, otherwise a Mann–Whitney *U* test (MW test) was performed. A *p*‐value smaller than 0.05 refers to (*), smaller than 0.01 to (**), smaller than 0.001 (***), and smaller than 0.0001 (****). Bottom and top of boxplots represent 25% and 75% percentile, respectively. Whiskers represent lower and upper quartile.

When we labeled motor axons under the control of the *vGlut* enhancer sequences [*OK371‐Gal4, UAS‐APEX2* ^*myr*^] in median, we identified 33 axons out of 76 axons per nerve. Since the summed median numbers of cholinergic (47) and glutamatergic (33) axons exceed the median total number of axons (78 or 76), we anticipate the existence of dual transmitting neurons. The median axonal area of glutamatergic axons is 0.067 μm^2^, corresponding to a radius of 0.15 μm (Figure 2E–G; magenta shading indicates glutamatergic neurons throughout the manuscript; see Table 1 for statistical analysis). As already noted for cholinergic axons, we did not observe large differences in the median axonal areas of individual animals (Figure 2H, magenta dots).

### 2.4 The Axonal Area Varies Along the Axonal Length

In summary, cholinergic axons are generally smaller when compared to glutamatergic axons. Interestingly, larger axon profiles often harbor mitochondria, whereas they are absent in small caliber axons (Figure 2C,D, white asterisks). This could indicate that the axonal diameter can vary along the length of an axon.

**FIGURE 3:**
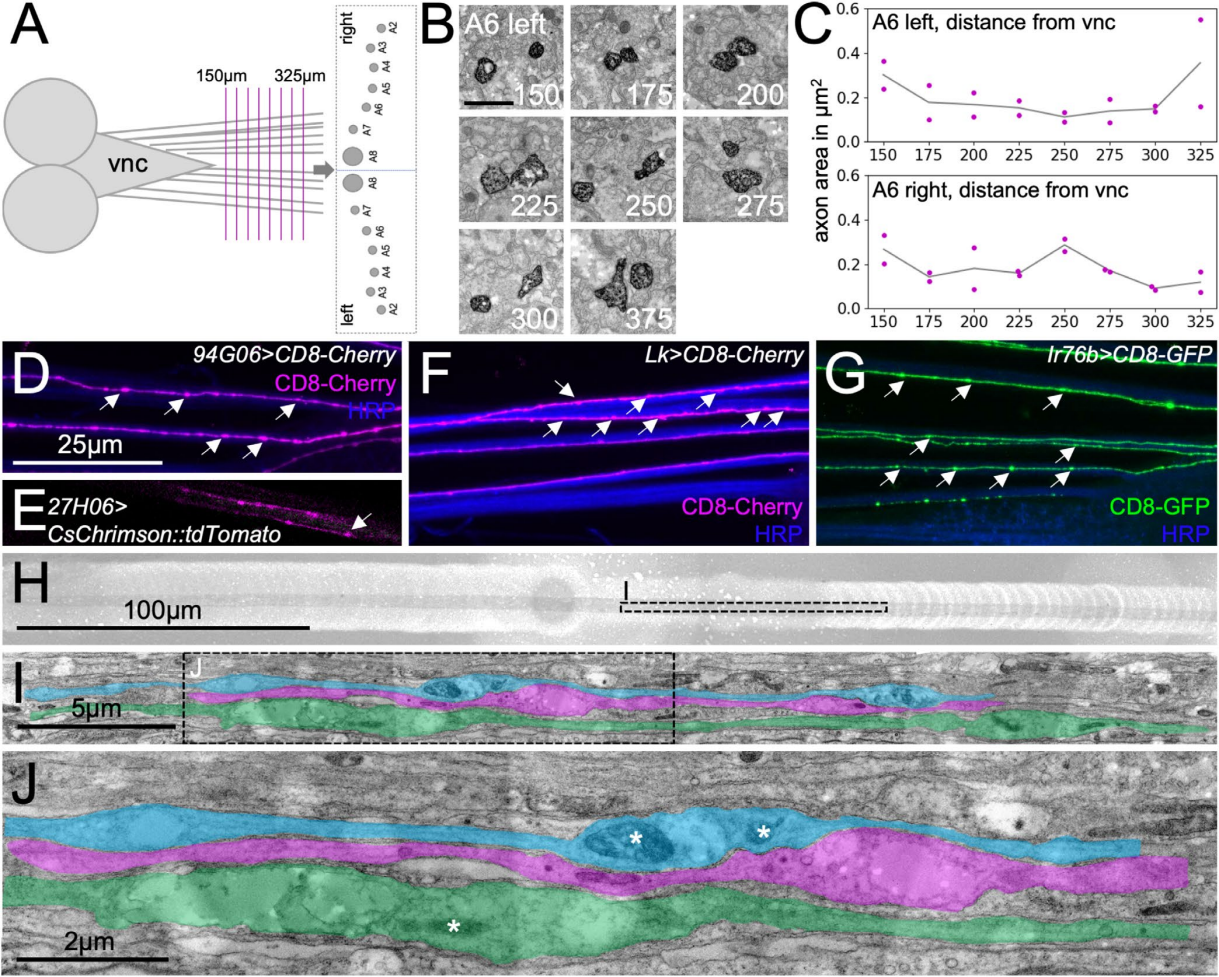
Axonal area changes along the axonal length. (A) Schematic representation of the sectioning mode. Sections were obtained every 25 μm from 150 to 325 μm distance from the tip of the ventral nerve cord. Nerve identity is deduced from its relative position. (B) Exemplary electron microscopic images of a left A6 nerve of a DAB‐stained animal of the genotype [*27E09‐Gal4*, *UAS‐APEX* ^*myr*^]. Scale bar is 1 μm, numbers indicate distance to the tip of the ventral nerve cord. (C) Changes in axonal area of the two APEX^myr^ positive axons in the left and right A6 nerves over the indicated distance to the ventral nerve cord. Single dots (magenta) indicate individual axon areas, gray line indicated the average area. (D–E) Confocal images of abdominal nerves from third instar larvae carrying the indicated Gal4 driver constructs that confer expression in small neuronal subsets. Note the frequent occurring varicosities (white arrows) that also occur in live preparations (E). Scale bar is 25 μm for all confocal images. (H) Overview of a longitudinal section through an abdominal nerve of more than 400 μm in length. The dotted area is shown in (I). (I) Three parallel running axons are indicated in false color to highlight varicosities. (J) Higher magnification of the boxed area in (I). Note the presence of mitochondria (white asterisks) in varicosities. Scale bars are as indicated.

To directly test this, we sectioned a larval filet preparation every 25 μm starting at a 150 μm distance from the tip of the ventral nerve cord (Figure 3A). Different nerve identities can be faithfully resolved in these preparations. When we analyzed the axonal area of the two *27E09‐Gal4* positive motor axons in identified nerves (Figure 3B, exemplary electron micrographs of a left A6 nerve), we noted variations in the axonal area along the length of the nerve analyzed (Figure 3C). In addition, we found nanoscopic varicosities repeatedly along the length of the axon when we observed single labeled axons of different modalities in the confocal microscope (Figure 3D–G). The axonal varicosities are also evident when axons are imaged in living larvae, indicating that they do not occur due to fixation artifacts (Figure 3E). Finally, we performed longitudinal sections of a single abdominal nerve, which revealed the presence of 2–3 μm long axonal varicosities often harboring mitochondria (Figure 3H–J).

In summary, Drosophila motor and sensory axons locate to distinct areas of the nerve, and motor axons are significantly larger in diameter compared to sensory axons. However, the diameter of a given axon can vary considerably due to varicosities that often harbor mitochondria. Interestingly, a similar observation was recently described for non‐myelinated CNS axons in the mouse brain (Griswold et al. 2025).

### 2.5 Wrapping of Sensory and Motor Axons

**FIGURE 4:**
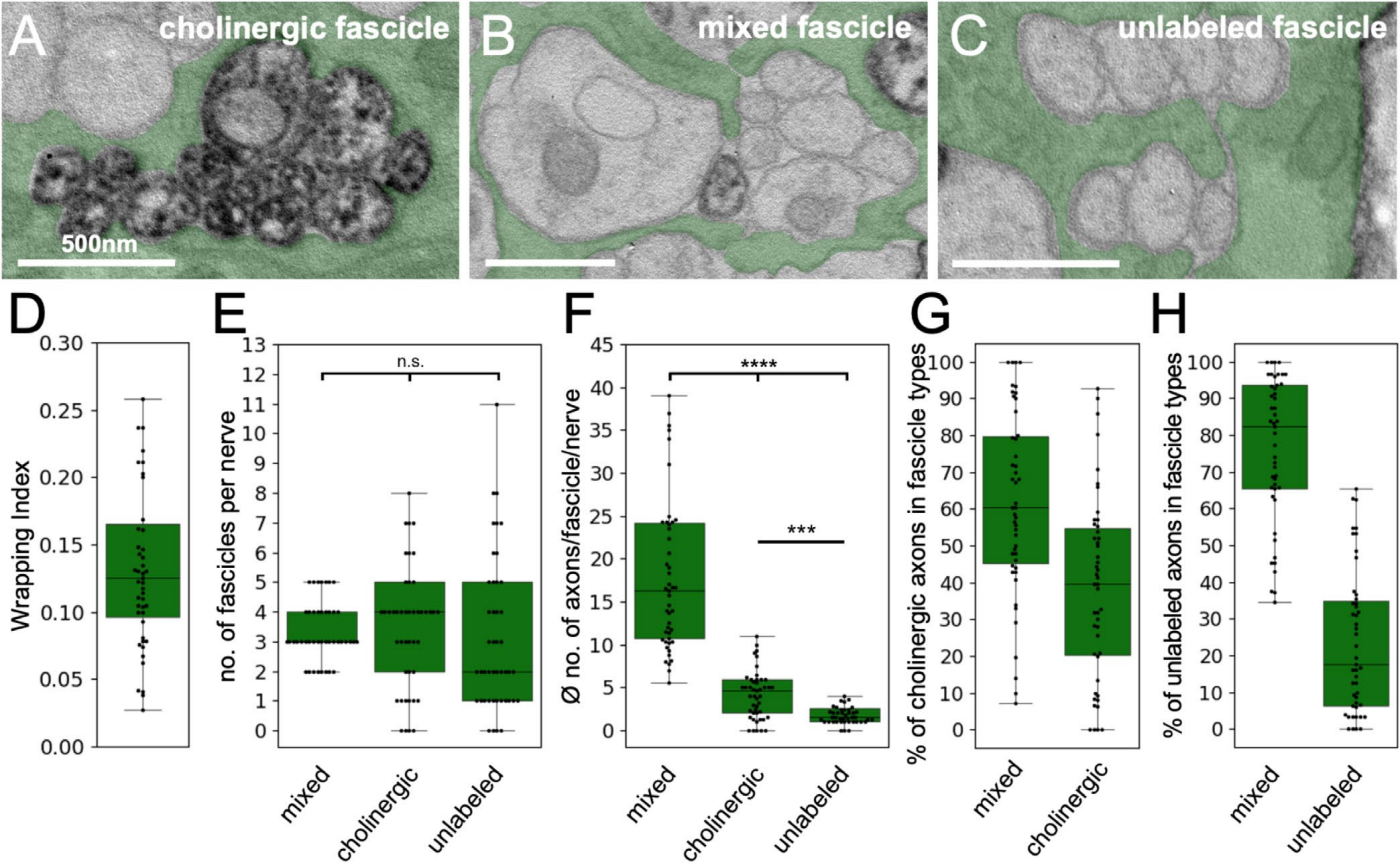
Differential glial wrapping of sensory and motor axons. (A–C) Exemplary pictures of the different wrapping modes found in third instar larva that express APEX^myr^ within cholinergic axons, therefore wrapping glia is pseudo‐colored in green. (A) Pure cholinergic, APEX2 positive fascicle, (B) mixed fascicle containing axons of both cholinergic as well as unlabeled axons of likely glutamatergic neurons, (C) fascicles harboring only unlabeled, likely glutamatergic axons. (D) Quantification of the overall wrapping index which is calculated by dividing the number of all axons by the number of fascicles. (E) The median number of the different fascicle types is indicated. No significant differences are found. (F) The median number of axons within the different fascicle types is significantly different. (G) Percentage of APEX2^myr^ expressing, cholinergic axons found in either mixed or pure cholinergic fascicles. (H) Percentage of unlabeled, mostly glutamatergic axons found in either mixed or pure fascicles. For details on the statistical analysis see Table 2.

**FIGURE 5:**
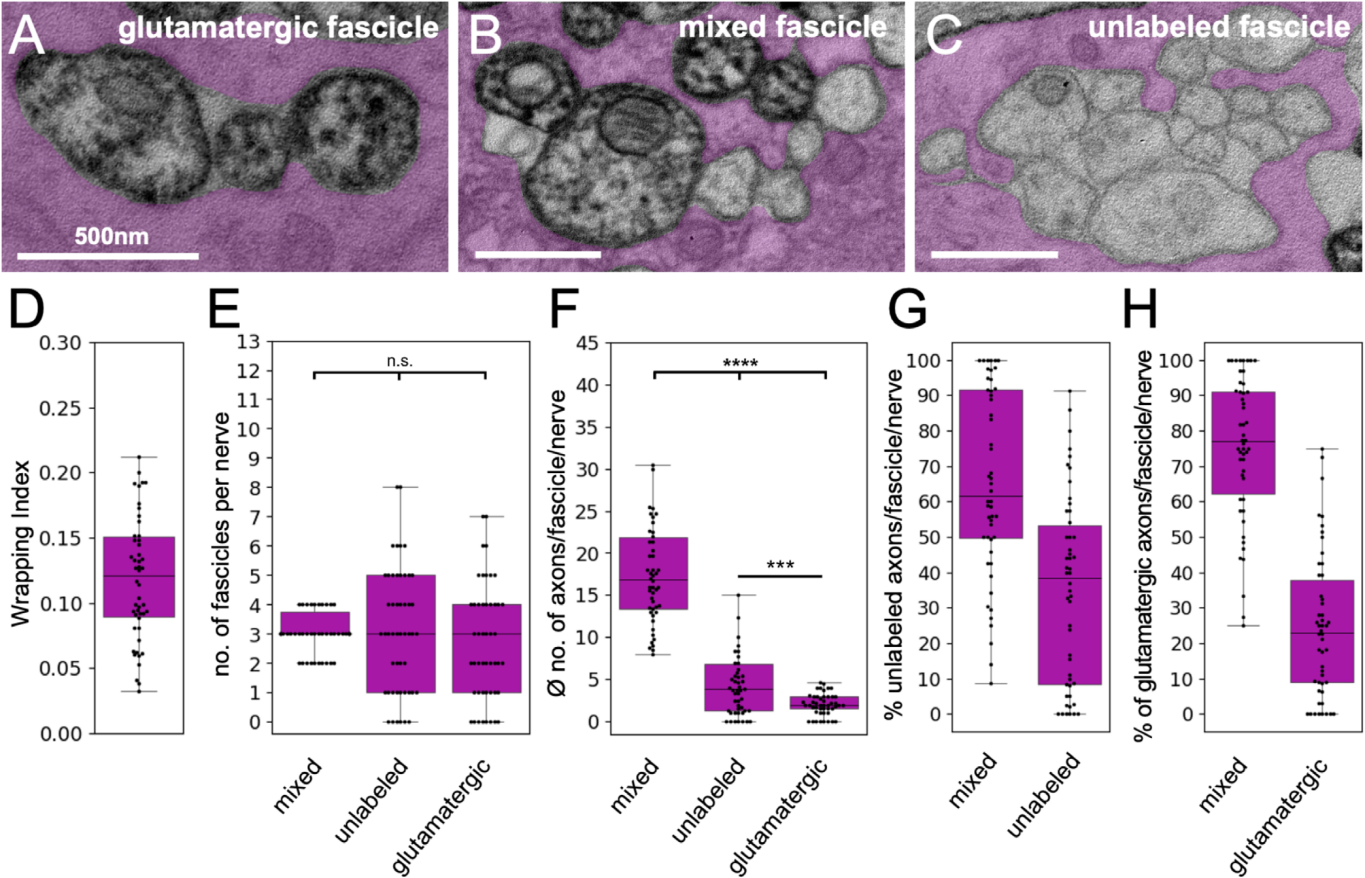
Differential glial wrapping of sensory and motor axons. (A–C) Exemplary pictures of the different wrapping modes found in third instar larva that express APEX^myr^ within glutamatergic axons, therefore wrapping glia is pseudo‐colored in magenta. (A) Pure glutamatergic, APEX2 positive, fascicle, (B) mixed fascicle containing axons of both unlabeled axons of likely cholinergic neurons as well as glutamatergic neurons, (C) fascicle harboring only unlabeled, likely cholinergic axons. (D) Quantification of the overall wrapping index. (E) The median number of the different fascicle types is indicated. No significant differences are found. (F) The median number of axons within the different fascicle types is significantly different. (G) Percentage of unlabeled, likely cholinergic axons found in either mixed or pure fascicles. (H) Percentage of labeled, glutamatergic axons found in either mixed or pure fascicles. For details on the statistical analysis see Table 3.

Given the organized arrangement of glutamatergic and cholinergic axons within the nerve, we next tested whether the wrapping glia differentially interact with axons of the two modalities. For this, we either labeled cholinergic (Figure 4) or glutamatergic axons (Figure 5) by expressing APEX2^myr^ and monitored their interaction with the wrapping glia. Generally, axons can be enwrapped in exclusive cholinergic or glutamatergic clusters as well as in mixed fascicles (Figures 4A–C and 5A–C).

To deduce the interaction of these fascicles with the wrapping glia, we determined the wrapping index, which is derived from the number of individually wrapped axons and fascicles divided by the number of all axons within the nerve (Matzat et al. 2015). We compared the wrapping indices of animals expressing CD8::GFP in all neurons with animals expressing APEX2^myr^ either in all cholinergic or in all glutamatergic neurons and found no significant difference (Figures S1, 4D, and 5D), see Tables 2 and 3 for statistical analysis). From this, we conclude that APEX2^myr^ expression does not affect the wrapping of axons; however, the position of sectioning along the anterior/posterior axis of the nerve might be of relevance. When we determined the wrapping index independently for nerves A3–A7 (left) and A2–A7 (right), we found an increase in the wrapping index in the shorter nerves (Figure S2).

**TABLE 2:** Statistics to Figure 4.

	WI	No. of fascicles per nerve	Ø no. of axons per fascicles	% of cholinergic axons/fascicle/nerve	% of unlabeled axons/fascicle/nerve
Animals	5				
Nerves	47				

		Mixed	Cholinergic	Unlabeled	Mixed	Cholinergic	Unlabeled	Cholinergic	Unlabeled	Cholinergic	Unlabeled
Median	0.125	3	4	2	16.33	4.6	1.5	60.42	39.58	82.35	17.65
Shapiro	0.0007	0.004	0.00015	7.32 × 10^−06^	1.1 × 10^−06^	0.00015	7.38 × 10^−08^	0.162		0.001	
*p*‐value	—	0.32		—	7.95 × 10^−05^		—	2.32 × 10^−05^		1.18 × 10^−16^	
		0.15			2.3 × 10^−16^						
		—	0.2		—	0.0002					

*Note*: Forty‐seven nerves from five animals expressing APEX2 in cholinergic neurons were analyzed. WI, wrapping index. The Shapiro test was used to test for normal distribution of the data. Upon normal distribution, a *t* test was used; otherwise, a Mann–Whitney *U* test (MW test) was performed. A *p* value smaller than 0.05 refers to (*), smaller than 0.01 to (**), smaller than 0.001 (***) and smaller than 0.0001 (****). Bottom of and top of boxplots represent the 25% and 75% percentiles, respectively. Whiskers represent the lower and upper quartiles.

**TABLE 3:** Statistics to Figure 5.

	WI	No. of fascicles per nerve	Ø no. of axons per fascicles	% of unlabeld axons/fascicle/nerve	% of glutamatergic axons/fascicle/nerve
Animals	5				
Nerves	50				

		Mixed	Unlabeled	Glutamatergic	Mixed	Unlabeled	Glutamatergic	Unlabeled	Glutamatergic	Unlabeled	Glutamatergic
Median	0.119	3	3	3	16.83	3.84	2	61.52	38.48	76.95	23.05
Shapiro	0.003	7.38 × 10^−05^	0.02	3.15 × 10^−05^	0.009	4.65 × 10^−06^	1.26 × 10^−06^	0.0039		0.0087	
*p*‐value	vs. ChAT 0.28	1			5.33 × 10^−14^		—	1.04 × 10^−06^		1.51 × 10^−07^	
		0.36			1.15 × 10^−17^						
		—	0.6		—	0.006					

*Note*: Fifty nerves from five animals expressing APEX2 in glutamatergic neurons were analyzed. The wrapping index is not significantly different from the wrapping index found in animals expressing APEX2 in cholinergic neurons. WI, wrapping index. The Shapiro test was used to test for normal distribution of the data. Upon normal distribution, a *t* test was used; otherwise, a Mann–Whitney *U* test (MW test) was performed. A *p*‐value smaller than 0.05 refers to (*), smaller than 0.01 to (**), smaller than 0.001 (***), and smaller than 0.0001 (****). Bottom of and top of boxplots represent the 25% and 75% percentiles, respectively. Whiskers represent the lower and upper quartiles.

Importantly, in these specimens, the A7 nerve was cut 150 μm distal to the CNS, while the outer nerves, such as the A3 nerve, were cut about 400 μm distal from their origin at the CNS (see also scheme in Figure 3A). Thus, axons either interact with glia in a segment‐specific manner or, alternatively, the wrapping index increases along the length of the nerve. To discriminate between these possibilities, we sectioned one larva with stained glutamatergic axons at different positions along the anterior/posterior axis and determined the specific wrapping indices. Here, we noted that, exemplary within the left A6 nerve, the wrapping index increases with distance from the CNS (Figure S3B′–E′,G). This was also the case in the majority of the other examined nerves (Figure S4). Possibly, wrapping glial cell growth around axons is initiated close to the wrapping glia nucleus and progressively extends to the proximal and distal ends. The zone close to the CNS is the least well‐wrapped area.

At the point of sectioning, 150 μm distal to the tip of the ventral nerve cord, the nerves contain a median of eight/nine fascicles. Three fascicles are made up of cholinergic and glutamatergic axons, three fascicles contain only cholinergic, and two/three contain only glutamatergic axons (Figures 4E and 5E, Tables 2 and 3). Importantly, the number of axons in the different fascicle types differs. In abdominal nerves, mixed fascicles contained by far the largest number of axons, followed by cholinergic fascicles, which still contained significantly more axons than pure glutamatergic fascicles (Figures 4F and 5F, Tables 2 and 3).

Finally, we wondered how many of the cholinergic and glutamatergic axons within peripheral nerves are enwrapped exclusively or in a mixed fashion. To address this, we calculated the percentage of cholinergic or glutamatergic axons that are exclusively wrapped in homotypic fascicles. When we labeled all cholinergic neurons [*ChaT‐Gal4, UAS‐APEX2* ^*myr*^] around 40% of the labeled axons are wrapped in exclusive cholinergic fascicles, which harbor a median of five axons. The remaining 60% of the cholinergic axons reside in large mixed fascicles (Figure 4G, Table 2). The unlabeled axons largely correspond to the axons of the glutamatergic neurons. Interestingly, only about 20% of the unlabeled axons are covered by wrapping glial cell processes without any contact with DAB‐labeled axons (Figure 4H, Table 2). Here, only three axons are wrapped by a single glial sheet. When all glutamatergic axons were labeled [*OK371‐Gal4, UAS‐APEX2* ^*myr*^] we noted a very similar distribution of the axons within the different fascicle types (Figure 5G,H, Table 3). This further indicates that APEX2^myr^ expression has no influence on the glial wrapping behavior.

In summary, glutamatergic axons are not only larger compared to cholinergic axons; they also seem to be more strongly associated with wrapping glia cells when they are exclusively wrapped, indicated by the significantly smaller fascicle size (Figures 4F and 5F).

### 2.6 Wrapping Glia Differentially Support Sensory and Motor Axons

Given that cholinergic and glutamatergic axons are wrapped differentially, we asked whether loss of wrapping glia affects sensory and motor axons to the same degree. For this, we established flies that allow *LexA‐dependent* ablation of wrapping glia by *hid* expression concomitantly with *Gal4*‐based expression of *APEX2* ^*myr*^ in the neurons of interest. We used a refined version of the transcriptional activator LexA that is repressible by Gal80 (Yagi et al. 2010). To specifically target the wrapping glia, we used *nrv2*‐*LexA* together with *90C03‐Gal80*, which directs expression of Gal80 in all CNS‐derived, *nrv2‐positive* glial cells (Kottmeier et al. 2020). The efficacy of LexA‐induced glial cell death was first assayed using the confocal microscope by adding a *LexAop‐CD8::GFP* element. In only one out of five larvae, we could detect some GFP‐positive remnants of wrapping glia in the posterior part of the A8 nerve (Figure S5A,B, arrows). In all other animals, the wrapping glia appear lost except for some small GFP‐positive puncta (Figure S5C, asterisks). In larvae with Gal4‐based wrapping glia ablation, remnants of glial cell processes can be seen in all posterior parts of the A8 nerves (Figure S5D–J). However, in all cases, no debris was detected in any nerve close to the CNS.

**FIGURE 6:**
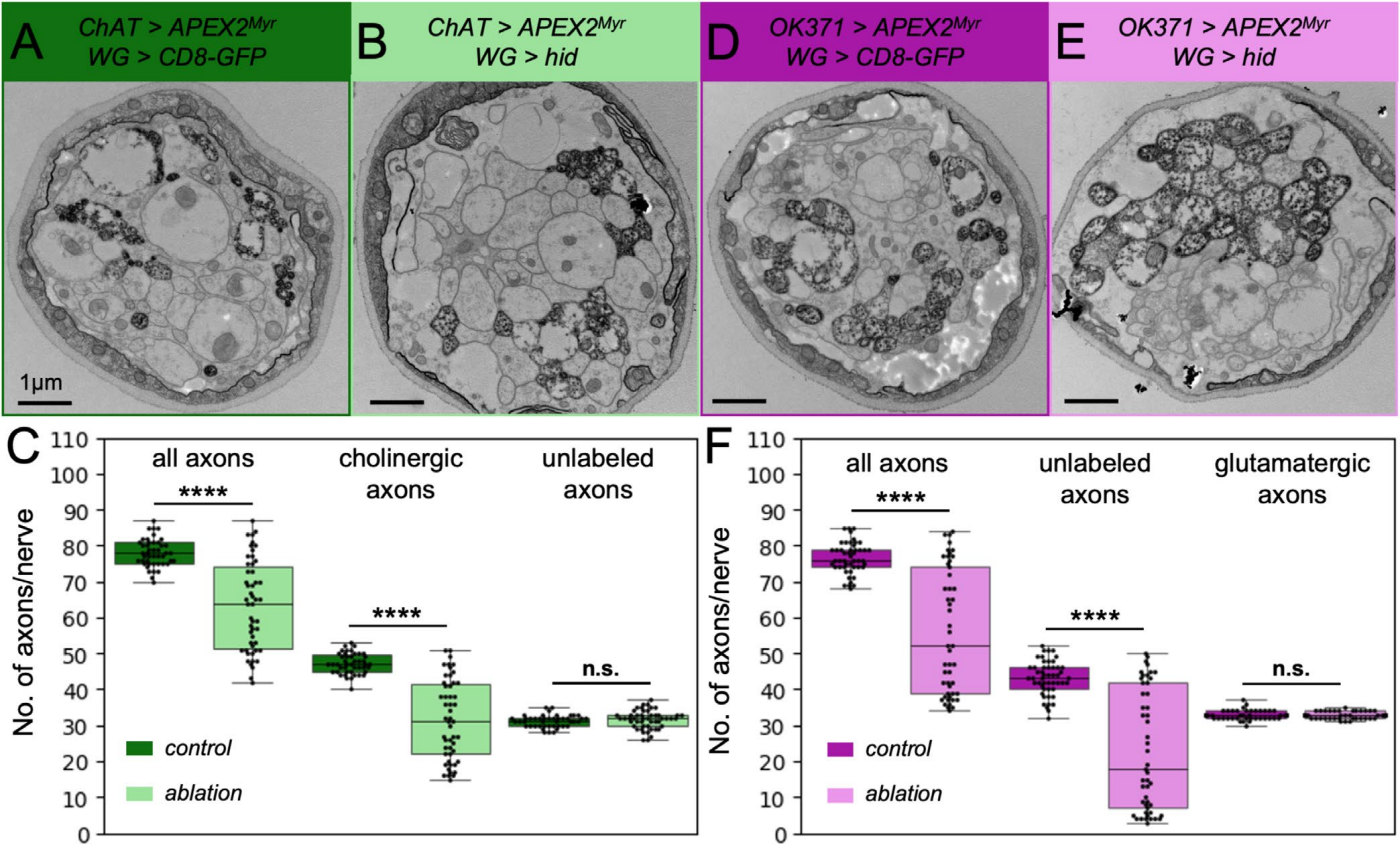
Wrapping glial ablation preferentially affects sensory axons. (A, B) Exemplary electron micrographs of cross sections through nerves in which cholinergic axons were stained either in the background of intact (A) as well as ablated wrapping glia (B). WG indicates the wrapping glia driver [*90C03‐Gal80; nrv2‐LexA*]. (C) Quantification of the number of all, cholinergic and unlabeled glutamatergic axons per nerve. In wrapping glia ablated animals, a significant reduction in the overall axon number can be observed. Only cholinergic axons appear affected while the number of unlabelled, likely glutamatergic axons stays constant. (D, E) Exemplary electron micrographs of cross sections through nerves in which glutamatergic axons were stained either in the background of intact (D) as well as ablated wrapping glia (E). The respective genotypes are indicated. (F) Quantification of the number of all, unlabeled cholinergic and glutamatergic axons per nerve. In wrapping glia ablated animals, a significant reduction in the overall axon number can be observed. Only the unlabeled, likely cholinergic axons are affected while the number of glutamatergic axons stays constant. For details on the statistical analysis see Table 4.

To investigate the effects of wrapping glia loss on cholinergic axons, we used LexA‐based ablation and established the following genotype [*Chat‐Gal4/90C03‐Gal80; UAS‐APEX2* ^*myr*^, *nrv2‐LexA/UAS‐APEX2* ^*myr*^, *LexAop‐hid*]. In addition, we generated a corresponding control carrying a *LexAop‐CD8::GFP* construct instead of a *LexAop‐hid* construct (Figure 6A,B). When we determined the median number of axons 150 μm distant from the tip of the ventral nerve cord in such wrapping glia‐ablated animals, we noted a significant reduction in their overall number. Counting of stained cholinergic axons and unstained axons separately revealed that axons of cholinergic neurons degenerate. In contrast, the number of the unlabeled axons, mainly corresponding to glutamatergic neurons, appeared to stay constant (Figure 6C, Table 4).

**TABLE 4:** Statistics to Figure 6 showing the number of axons per nerve.

	Figure 6C	*ChAT>APEX2* ^*myr*^	Figure 6F	*OK371 > APEX2* ^*myr*^
	Control	Ablation	Control	Ablation
Animals	5	5	5	5
Nerves	47	51	50	49

	No. of axons/nerve											
	All		Cholinergic		Unlabeled		All		Unlabeled		Glutamatergic	
	Contr.	Abl.	Contr.	Abl.	Contr.	Abl.	Contr.	Abl.	Contr.	Abl.	Contr.	Abl.
Median	78	64	47	31	31	32	76	52	43	18	33	33
Shapiro	0.2	0.04	0.018	0.011	0.46	0.07	0.32	0.0001	0.047	5.04 × 10^−05^	0.0009	5.04 × 10^−08^
*p*‐value	2.6 × 10^−11^		—		—		3.71 × 10^−08^		—		—	
	—		1.18 × 10^−11^		—		—		2.86 × 10^−11^		—	
	—		—		0.4		—		—		0.8	

*Note*: The Shapiro test was used to test for normal distribution of the data. Upon normal distribution, a *t* test was used; otherwise, a Mann–Whitney *U* test (MW test) was performed. A *p*‐value smaller than 0.05 refers to (*), smaller than 0.01 to (**), smaller than 0.001 (***), and smaller than 0.0001 (****). Bottom of and top of boxplots represent the 25% and 75% percentiles, respectively. Whiskers represent the lower and upper quartiles.

**TABLE 5:** Statistics for Figure 7, showing the median number of sensory and motor axons in identified nerves sectioned 150 μm from the CNS.

Figure 7B	*ChAT‐Gal4 > APEX2* ^*myr*^, *nrv2‐LexA, 90C03‐Gal80 > hid*											
	Left						Right					
	A2	A3	A4	A5	A6	A7	A7	A6	A5	A4	A3	A2
Nerves	1	3	5	5	5	5	5	5	5	5	5	2
Median of all axons	67.0	79.0	70.0	57.0	50.0	52.0	48.0	53.0	60.0	75.0	77.0	78.5
Median of cholinergic axons	38.0	46.0	38.0	27.0	24.0	22.0	17.0	23.0	27.0	42.0	44.0	47.0
Median of unlabeled axons	29.0	32.0	32.0	31.0	31.0	32.0	32.0	32.0	33.0	33.0	33.0	31.5

Figure 7C	*Ok371‐Gal4 > APEX2* ^*myr*^, *nrv2‐LexA, 90C03‐Gal80 > hid*											
	Left						Right					
	A2	A3	A4	A5	A6	A7	A7	A6	A5	A4	A3	A2
Nerves	1	3	4	5	5	5	5	5	5	5	5	1
Median of all axons	81.0	72.0	74.5	38.0	37.0	42.0	42.0	39.0	52.0	51.0	76.0	77.0
Median of unlabeled axons	48	39	40.5	10	4	9	8	7	19	17	42	44
Median of glutamatergic axons	33	32	33.5	33	33	33	33	32	33	34	33	33

We next expressed *APEX2* ^*myr*^ in all glutamatergic neurons in either control or wrapping glia ablated animals using the following genotype: [*OK371‐Gal4/90C03‐Gal80; UAS‐APEX2* ^*myr*^, *nrv2‐LexA/UAS‐APEX2* ^*myr*^, *LexAop‐hid*] (Figure 6D,E). Here, we again noted a significant reduction in the median overall number of axons. The unlabeled axons, mainly corresponding to cholinergic neurons, were significantly reduced in number. The number of stained glutamatergic axons did not significantly change (Figure 6F, Table 4).

Interestingly, the reduction in the number of cholinergic axons differed in the two experiments (Figure 6C,F). To test whether this difference might be due to differential ablation efficacy, we determined the number of nerve profiles lacking any wrapping glia remnants. The ablation of wrapping glia appeared slightly more efficient in animals expressing APEX2^myr^ in glutamatergic neurons compared to animals with APEX2^myr^ expressing cholinergic neurons (Figure S6G–K). This suggests that the reduction in the number of cholinergic axons correlates with the efficacy of glial ablation (Figure 6C,F). In conclusion, we demonstrate that the loss of wrapping glia primarily affects the cholinergic axons and that the expression of *APEX2* ^*myr*^ has no effect on the differential survival of cholinergic and glutamatergic axons.

We further wondered whether wrapping glia ablation also affects other aspects of axon morphology and analyzed the axonal area of cholinergic and glutamatergic axons in control and wrapping glia ablated animals. Surprisingly, both cholinergic as well as glutamatergic axons increase their median diameter upon wrapping glia ablation (Figure S6A). In the case of cholinergic axons, small diameter axons might degenerate first and larger caliber axons remain preferentially. However, this is not the case for glutamatergic axons. This suggests that the increase in axonal diameter is an axon intrinsic mechanism triggered by wrapping glia ablation. By further analyzing the axonal area of glutamatergic axons in a nerve‐specific manner, we could show that this mechanism is not affected by nerve identity (Figure S6B).

### 2.7 Sensory Axons Show a Length‐Dependent Requirement for Glial Support

In the above experiments, we found nerves with a reduced number of sensory axons but also nerves that carried the normal number of axons. We, therefore, wondered whether the observed loss of axons may depend on the segmental origin of the nerve. In order to address this question, we carefully prepared, fixed, stained, and sectioned larval filets to be able to identify the different nerves according to their position above the musculature. The A8 nerve always occupies the medial most position in these preparations. It can be further identified by its larger number of axons (Matzat et al. 2015) and was therefore not included in the subsequent analysis. Next to the A8 nerves, the abdominal nerves A7–A2 occupy increasingly more lateral positions (Figures 3A, S7, and S8). The thoracic nerves, as well as the A1 nerve, are not visible at the plane of section 150 μm distal from the tip of the ventral nerve cord.

**FIGURE 7:**
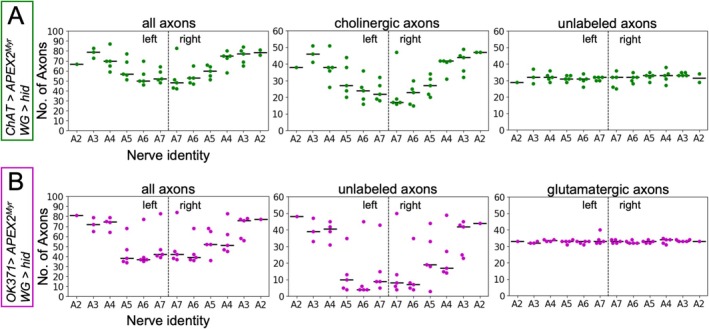
Loss of cholinergic axons in individual nerves at 150 μm distance from the ventral nerve cord. WG indicates the wrapping glia driver [*90C03‐Gal80; nrv2‐LexA*]. (A) Number of all axons, cholinergic axons, and unlabeled axons in identified nerves of animals with stained cholinergic axons in a wrapping glia ablated background (*n* = 5 animals). Green dots represent the number of axons within individual nerves of different animals. The median number of axons is plotted as black line (for statistical analysis see Table 5). Axonal loss is restricted to cholinergic axons while the number of unlabeled axons stays constant. (B) Same analysis as in (A) but glutamatergic axons were stained instead (for statistical analysis see Table 5).

Knowing the nerve identity, it became apparent that the median number of axons in very long nerves (A5–A7) is lower compared to the number of axons in the abdominal nerves innervating segments A2–A4. Furthermore, axonal loss is restricted to cholinergic axons, while the number of unlabeled, glutamatergic axons appears rather constant in the abdominal nerves A2–A7 (Figure 7A). Very similar results were obtained from third instar larval filet preparations when glutamatergic axons were labeled. Here, degeneration of unlabeled cholinergic axons also appeared to be stronger in the very long nerves (Figure 7B).

**FIGURE 8:**
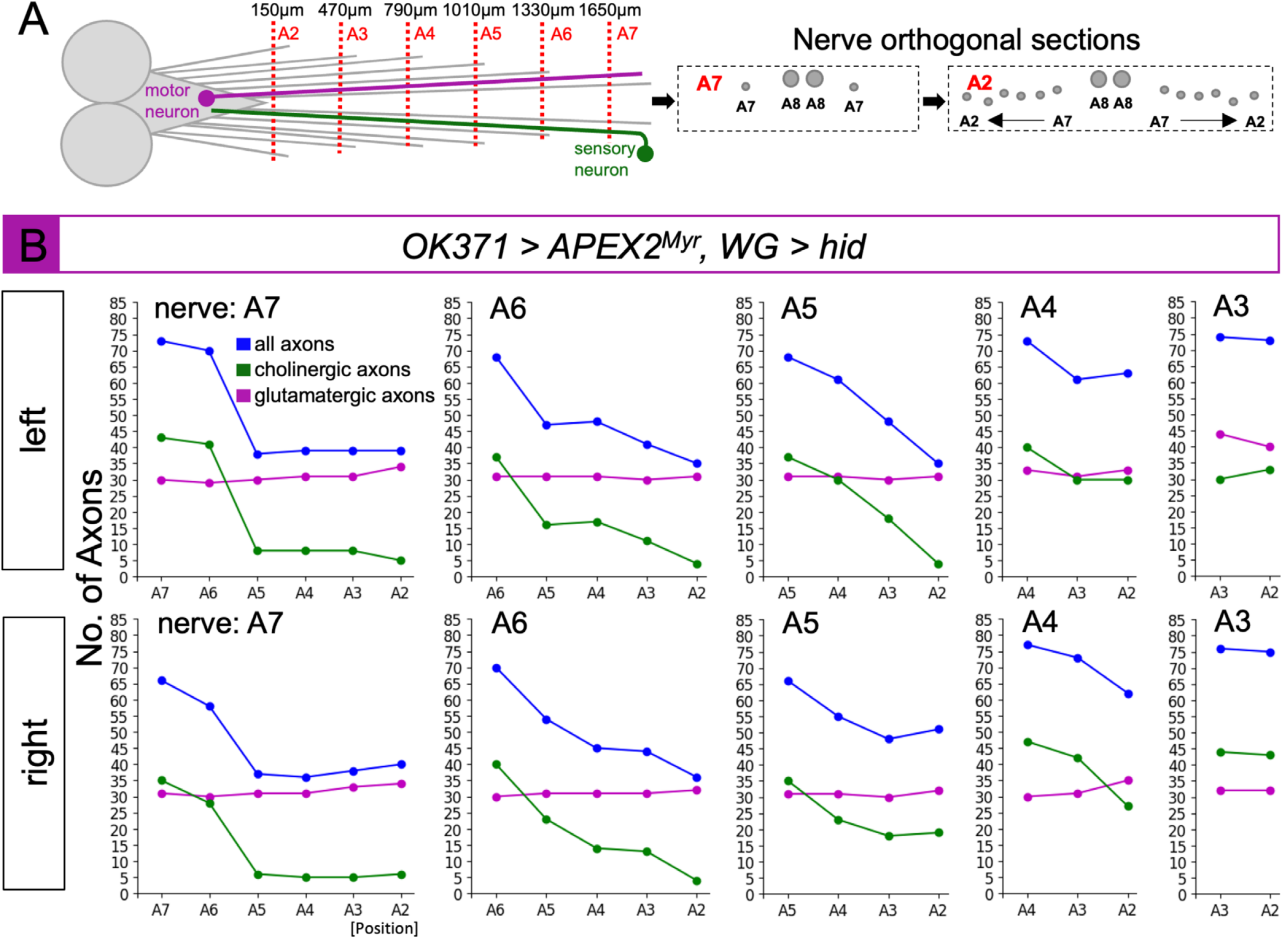
Length dependent degeneration of cholinergic axons. WG indicates the wrapping glia driver [*90C03‐Gal80; nrv2‐LexA*]. (A) Scheme of a larval filet. The positions of sectioning are indicated. In the A7 segment only abdominal nerves A7 and A8 are present, whereas in the A2 segment abdominal nerves A2–A8 can be detected. (B) Glutamatergic axons were stained in a wrapping glia ablated larva. The number of axons was counted and plotted against the segment in which the nerve was cut. In all nerves the number of axons drops within a distance of 400–600 μm which corresponds to approximately two segments. The reduction in the number of axons is mainly attributed to a loss of cholinergic axons, while the number of glutamatergic axons stays constant.

The above data suggests that longer sensory axons are most sensitive to the loss of wrapping glia. However, a length‐dependent degeneration of motor axons would have escaped our analysis. This might be due to the origin of motor axons that leave the ventral nerve cord not far from the section plane. We, therefore, sectioned a third instar larval filet of the following genotype [*OK371‐Gal4/90C03‐Gal80; UAS‐APEX2* ^*myr*^, *nrv2‐LexA/UAS‐APEX2* ^*myr*^, *LexAop‐hid*], in which all glutamatergic axons are labeled in the absence of wrapping glia. We performed “serial sectioning” from posterior to anterior at six distinct positions corresponding to the segments A7–A2 (Figure 8A). This allowed us to trace the nerves and determine the number of stained glutamatergic motor and unstained sensory axons in each of the segments of a single animal (Figures 8B, 9, and S10).

We found that the number of cholinergic axons in a given nerve drops over the distance of two segments. In the A7 nerve, most of the cholinergic axons can still be detected at a position in segments A7 and A6. However, their number drops significantly in segment A5. In all other nerves, except A2 and A3, a similar loss of cholinergic axons can be detected two to three segments anterior to their origin (Figure 8B). This suggests that wrapping glial cell contact is needed to sustain the survival of sensory axons approximately 400–600 μm distal to the neuronal cell body. In contrast, as the number of glutamatergic axons stays rather constant over the entire length of the third instar larva, these axons do not appear to rely on trophic support by the wrapping glial cells to the same extent as sensory axons do.

## 3 Discussion

Here, we examined the organization of sensory and motor axons in abdominal nerves of third‐instar Drosophila larvae and investigated their differential interaction with wrapping glial cell processes. Within each nerve, axons of different neuronal modalities are spatially separated and are organized in a stereotyped fashion. Motor axons have a greater diameter than sensory axons and are more likely to be individually wrapped by wrapping glial cell processes. In contrast to motor axons, sensory axons crucially depend on glial support and show a length‐dependent degeneration in the absence of wrapping glia.

In the past, the identification of specific axons using electron microscopy has been difficult. The introduction of peroxidase‐based (HRP or APEX) labeling techniques, together with the ability to target the expression of these enzymes to specific neurons, now allows for the reliable detection of axons of interest. Moreover, in principle, double labeling approaches could be adopted for the electron microscope to, for example, relate the localization of *Tdc2* and *27E09* positive axons to each other. We therefore expressed a Tau::APEX2 fusion protein, which we expected to localize to microtubules, thus generating a labeling pattern distinct from the APEX2^myr^ pattern. Unfortunately, neither the Tau::APEX2 fusion nor a previously generated mCD8::GFP::APEX2 fusion protein (Tsang et al. 2018) resulted in differential labeling patterns of axons, possibly due to the relatively small volume of axons (Figure 11). Additionally, we noted that a cytosolic APEX2 version failed to localize to the axon (data not shown).

The robust APEX2^myr^ mediated axonal labeling demonstrated that motor axons in Drosophila peripheral nerves have larger diameters compared to axons of sensory neurons. This size difference is not obvious when looking at embryonic nerves (Stork et al. 2008) suggesting that differential growth is required. This may be linked to physiological needs, as the axonal diameter is proportional to the action potential conduction velocity. In addition, it has been demonstrated for vertebrate axons that the firing rate correlates with the axonal diameter, suggesting that high firing rates favor large diameters (Perge et al. 2012). Similar to vertebrates, where the diameter of an axon correlates to its target size, the larger diameter of Drosophila motor axons may also depend on the large size of their neuromuscular junctions (Cheng et al. 2011; Innocenti et al. 2014).

In the vertebrate PNS, the axonal diameter is a key driver in myelination (Feltri et al. 2016; Harty and Monk 2017; Nave and Werner 2014; Taveggia 2016). Large caliber axons are preferentially myelinated and thus metabolically separated from the environment, supporting the finding that they require metabolic support from the myelinating glia (Fünfschilling et al. 2012; Lee et al. 2012; Nave 2010). Smaller axons (< 0.1 μm) that are usually sensory or autonomic axons are preferentially wrapped in Remak fibers in which several axons or small fascicles are engulfed (Kidd et al. 2013). Here, we could show that the smaller sensory axons are preferentially enwrapped in larger, homotypic fascicles, while the larger motor axons tend to be enwrapped together with axons of a different modality. However, if they are exclusively enwrapped, they are found in very small fascicles. This could indicate that the axon–axon adhesion is more pronounced between sensory axons and that glia–sensory axon interactions are not as strong as the glia‐motor axon interactions (Baldenius et al. 2023). In consequence, we expected that large motor axons are more vulnerable to the loss of wrapping. Surprisingly, however, in the absence of wrapping glia, we found a length‐dependent degeneration of only the cholinergic sensory axons while larger glutamatergic motor axons remained unaffected. The L1 wing nerve of adult flies harbors 290 sensory neurons, of which about 40 are glutamatergic (Neukomm et al. 2014). Upon ablation of the wrapping glia in the adult wing, age‐dependent axon degeneration can be detected after 14 days, but it is unknown whether cholinergic and glutamatergic neurons behave differentially (Lassetter et al. 2023).

Here we report on LexA‐mediated ablation of wrapping glia, which, based on the confocal analysis, appears to be more effective compared to Gal4‐mediated ablation. Moreover, LexA‐mediated ablation efficacy varies slightly among the different experiments, which can be deduced from the amount of remaining glial cell debris in abdominal nerves. Interestingly, we noted a correlation of these minor changes in ablation efficacy with the survival probability of sensory neurons. The stronger the ablation is, the more length‐dependent degeneration of cholinergic axons is observed in longer nerves (Figures 6 and 7). Under the condition of a weaker, Gal4‐mediated wrapping glia ablation, length‐dependent axonal degeneration has not been found. Similar axonal phenotypes were noted upon expression of a dominant negative Heartless receptor, further suggesting that Gal4‐mediated ablation was not as efficient as LexA‐mediated ablation and could account for the differential experimental outcomes (Kottmeier et al. 2020).

In larval nerves, the glial requirement for cholinergic axons becomes obvious only two segments distal to the cell body, suggesting that metabolic flux within thin axons cannot be sustained over long distances. Organelle and vesicle transport, as well as ion homeostasis due to action potential generation, require a constant supply of ATP along the axons. When ATP production was calculated for axons of different diameters in relation to action potential firing and resting potential maintenance, it was found that only large‐diameter axons (> 0.9 μm) are able to cover their own metabolic needs (Harris and Attwell 2012). Smaller‐diameter axons, in contrast, display a shortfall in ATP production, which makes metabolic support by glia necessary for those axons (Harris and Attwell 2012). In the absence of glial wrapping, metabolites or even ATP itself would have to passively diffuse through the axons to reach regions with high energy demand. However, in small‐diameter axons, diffusion may be hindered by the dimensions of the axon, and thus small‐caliber axons are more likely to degenerate compared to the larger motor axons. Such a differential diffusion in differentially sized axons is well known and is the basis for diffusion MRI imaging (Harkins et al. 2021). Dysfunctional glia also impact axonal morphology and generally result in axonal swelling, which is a hallmark of degenerating axons (Bhattacharya et al. 2012; Mire et al. 1970; Sodders et al. 2025). Similar to this, we could also detect an increase in the axonal diameter of cholinergic as well as glutamatergic axons upon wrapping glia ablation in our experiments.

In vertebrates, damage to peripheral glial cells leads to a highly complex group of diseases called peripheral neuropathies. In diabetic or chemotherapy‐induced neuropathies, Schwann cell function is impaired, accompanied by axonal damage and sensory symptoms are often predominant (Elbaz et al. 2022; Gonçalves et al. 2017; Imai et al. 2017). In a mouse model for peripheral neuropathy, different neuronal subtypes, such as sensory and motor axons, respond differentially to a loss of glial ensheathment (Elbaz et al. 2022). Moreover, feeding Drosophila larvae with paclitaxel, a chemotherapeutic agent that causes neuropathy in humans, causes a loss of axons within peripheral nerves (Bhattacharya et al. 2012). Since neuromuscular junctions were unaffected, it was speculated that paclitaxel primarily affects sensory axons. This phenotype is comparable to what we observed following wrapping glia ablation. Unfortunately, the authors did not explicitly examine wrapping glial cells in their study, but based on the published electron microscopic images, an impairment of their differentiation can be anticipated. Thus, the primary target of paclitaxel could be glial cells, and axonal loss is a secondary effect of the treatment and would likely be length dependent.

In conclusion, our study demonstrates that key aspects of nervous system function, in particular the interdependency of axons and glia, are conserved between vertebrates and invertebrates. Upon wrapping glia ablation, we observed a selective loss of sensory axons, suggesting that in the future, Drosophila models can contribute to further studies of peripheral sensory neuropathies in which glial function is disrupted, leading to axonal damage.

## 4 Materials and Methods

### 4.1 Fly Strains and Husbandry

Fly stocks were kept at room temperature on standard Drosophila food. All crosses for conducting experiments were raised at 25°C. All flies used in this study are listed in the table below.

**Table:** 

Fly strain	Description	Bloomington identifier/reference
*Tdc2‐Gal4*	Gal4 expression in octopaminergic/tyraminergic neurons	RRID:BDSC_9313/(Cole et al. 2005)
*27E09‐Gal4*	Gal4 expression in two motor neurons per abdominal hemineuromer	RRID:BDSC_49227 (Jenett et al. 2012)
*Ok371‐Gal4*	Gal4 expression in glutamatergic neurons	RRID:BDSC_26160/(Mahr and Aberle 2006)
*ChAT‐Gal4*	Gal4 expression in cholinergic neurons	RRID:BDSC_6793/(Salvaterra and Kitamoto 2001)
*ChAT‐LexA*	LexA^DBD^::QF^AD^ expression in cholinergic neurons	RRID:BDSC_60319/(Diao et al. 2015) 84379/(Deng et al. 2019)
*LexAop‐CD8::GFP*	LexA‐dependent expression of membrane tagged GFP	RRID:BDSC_32203/(Pfeiffer et al. 2010)
*UAS‐mCD8::GFP::APEX*	Gal4‐dependent expression of membrane bound mCD8::GFP::APEX2	RRID:BDSC_79626/(Tsang et al. 2018)
*UAS‐APEX2* ^*myr*^	Gal4‐dependent expression of myristoylated APEX2	(Rey et al. 2023)
*90C03‐Gal80*	Gal80 expression under control of the *90C03* enhancer	RRID:BDSC_602898/(Kottmeier et al. 2020)
*UAS‐Tau::APEX2*	Expresses a Tau::APEX2 fusion to target APEX2 to microtubules	This study
*nrv2‐LexA* ^*Gal4‐AD*^	Drives expression of a LexA::Gal4^AD^ fusion under control of the nrv2 enhancer	This study
*LexAop‐hid*	Expresses proapoptotic hid for targeted cell ablation	Kind gift of A. Bergmann, Worcester, MA, USA

### 4.2 Molecular Work

To generate *nrv2‐LexA* ^*Gal4‐AD*^ flies, a 3.6 kb fragment of the *nrv2* promoter genomic position: 6,796,598..6,800,207) was cloned into the pENTR/D‐Topo vector (Invitrogen, using primer: 5′‐CACCTTAGCCACCGACTCTGGTC‐3′, 5′‐AGGGGTATGGATATGTGAGGTG‐3′) and then transferred into pBPnlsLexA‐GADflUw (addgene #26232). To generate *LexAop‐tau::FLAG::Apex2‐NES* flies, FLAG‐APEX2‐NES DNA (addgene #92158) was inserted in frame 3′ to the Drosophila *tau* coding sequence using cold fusion (Biozol, Eching, Germnay, forward primer: 5′‐CAAGAAGAGAACTCTGAATAATGGCGGATGTCCTGGAG‐3′, reverse primer: 5′‐GACCTCGAGCCGCGGCCGCAGCTTTGTTGATTTAAATTTTCATCAGCG‐3′) in a pLexAopTattB‐rfa backbone (kind gift of S. Schirmeier, Dresden). The different DNA constructs were inserted into the *attp2* landing site using established protocols (Bischof et al. 2007; Groth et al. 2004; Pfeiffer et al. 2010).

### 4.3 Histology

For light microscopy, filets of third instar larvae were prepared in phosphate buffered saline (PBS). Subsequently, they were fixed with Bouin's solution for 3 min followed by three washing steps in PBS, 0.3% Triton‐X‐100 (PBT) each for 20 min. Samples were blocked in 10% goat serum in PBT for 1 h at room temperature. Incubation with primary as well as secondary antibody was done at 4°C overnight and separated by three washing steps in PBT each 20 min. Antibodies are listed in the table below. After antibody incubation, filets were covered with VECTASHIELD mounting solution (Vector Laboratories) and imaged using a Zeiss LSM880 Airyscan confocal microscope (Carl Zeiss AG).

**Table:** 

Antibody	Source or reference	Identifiers	Concentration
Anti‐GFP (rabbit, polyclonal)	Invitrogen	Cat#ab13970, RRID:AB_300798	1:1000
Anti‐rabbit Alexa 488 (goat, polyclonal)	Thermo Fisher	Cat#A‐11008, RRID:AB_143165	1:1000
Anti‐HRP Alexa647 (goat, polyclonal)	Thermo Fisher	Cat#31460, RRID:AB_228341	1:500

### 4.4 Sample Preparation and EM Imaging

For electron microscopy, filets of third instar larvae were prepared in 4% formaldehyde (FA) in 0.1 M phosphate buffer (P‐buffer) and prefixed for 45 min. Subsequently, filets were washed 5× with P‐buffer and incubated in P‐buffer containing 0.02 M glycine for 20 min. Filets were again washed 5× with P‐Buffer and incubated in 0.05% DAB in P‐Buffer for 40 min at room temperature. 0.03% H_2_O_2_ was added for 5 min to start the APEX2‐catalyzed DAB polymerization. Filets were washed 3× with P‐buffer and were fixed overnight in 4% FA plus 0.5% glutaraldehyde at room temperature. FA was replaced by 2% OsO_4_ in 0.1 M P‐Buffer for 1 h, followed by an en‐bloc uranyl acetate (UA) staining for 30 min using 2% UA in H_2_O at room temperature in the dark. Subsequently, an EtOH series (50%, 60%,70%,80%, 90%, and 96%) was performed on ice with 15 min for each step. Final dehydration was done at RT with 3× 100% EtOH, dehydrated using a molecular sieve and 2× propylene oxide for 15 min. After slow epon infiltration, filets were ultra‐flat embedded in gene frames (thermo scientific) between two ACLAR film layers and polymerized at 40°C for at least 3 days.

Ultrathin sections were taken at an ultramicrotome (Leica EM UC7) using a 35° ultra‐knife (Diatome) and collected on formvar‐coated one‐slot copper grids. Sections were imaged using an upgraded Zeiss TEM 900 (point electronics) at 80 kV and an iTEM software‐operated Morada camera (EMSIS, Münster, Germany).

### 4.5 Image Analysis and Statistics

Images were analyzed using standard functions of the ImageJ software (Fiji) (Schindelin et al. 2012). For statistical analysis, Visual studio code (Microsoft) with the python software extension v2024.14.1 was used.

## Author Contributions

S.K. performed all experiments, analysis of data and contribtuted to manuscript writing. S.R. generated APEX2myr expressing transgenes, generated nrv2‐lexA flies, supported EM work. A.K. Cloned APEX2 variants. C.K. secured funding, wrote the manuscript, conceptual input.

## Ethics Statement

All experiments were conducted according to the regulations of German law.

## Conflicts of Interest

The authors declare no conflicts of interest.

## Supporting information


**Figure S1.** Neuronal expression of APEX2^myr^ does not affect axon wrapping. (A) Exemplary cross section of an abdominal nerve of an animal with the genotype [*nsyb‐Gal4 UAS‐GFP*]. Scale bar is 1 μm. (B) Box plot showing the distribution of wrapping indices of individual nerves from control larvae [nsyb‐Gal4 UAS‐GFP] and larvae expressing APEX2^myr^ either in cholinergic [*ChAT‐Gal4 UAS‐APEX2* ^*myr*^] or in glutamatergic neurons [*OK371‐Gal4 UAS‐APEX2* ^*myr*^]. WG indicates the wrapping glia driver [*90C03‐Gal80; nrv2‐LexA*]. The median is indicated. (C) Statistics to (B). The numbers of analyzed animals and nerves is indicated. The Shapiro test was used to test for normal distribution of the data. Upon normal distribution a *t* test was used, otherwise a Mann–Whitney *U* test (MW test) was performed. Bottom and top of boxplots represent 25% and 75% percentile, respectively. Whiskers represent lower and upper quartile.

**Figure S2.** The wrapping index at 150 μm distance from the ventral nerve cord correlates with the nerve identity. (A) Wrapping index of individual left and right abdominal nerves determined for animals that had labeled cholinergic axons (green dots) or glutamatergic axons (magenta dots). The abdominal nerves A3 and A4 are sectioned further distant to the exit point at the ventral nerve cord. Only the A8 nerve originates 150 μm away from the section plane. WG indicates the wrapping glia driver [*90C03‐Gal80; nrv2‐LexA*]. (B) Statistics to (A). The median is calculated based on all data points.

**Figure S3.** The wrapping index changes over nerve length. WG indicates the wrapping glia driver [*90C03‐Gal80; nrv2‐LexA*]. (A) Light microscopic image of resin embedded, osmium stained third instar larval filet of the indicated genotype used for subsequent sectioning (B–F). (B–F) Overview electron microscopic images of sections taken at the positions indicated in (A). (B′–E′) High magnification images of the left A6 nerve showing the number of distinct fascicles indicated in false color (magenta). Note, that the glutamatergic motor axons are stained following APEX2^myr^ expression and cluster to one side of the nerve. (G) The wrapping index of the left abdominal nerve A6 changes with sectioning position (see Figure S4 for remaining nerves).

**Figure S4.** The wrapping index changes over nerve length. The data are obtained from the same animal as in Figure S3. The wrapping index of abdominal nerves A4–A7 is plotted separately over the segmental position.

**Figure S5.** Ablation efficacy. (A–F) Comparison of LexA and Gal4 mediated wrapping glia ablation (WG^LexA^ or WG^Gal4^). All neuronal membranes are stained using anti‐HRP antibodies (blue), GFP expression is shown in white. (A) Control larva expressing GFP in wrapping glia with the genotype [*nrv2‐LexA, 90C03‐Gal80; LexAop‐CD8::GFP*]. Note all wrapping glia cover the segmental nerves. (B, C) Upon ablation of the wrapping glia in animals with the genotype [*nrv2‐LexA, 90C03‐Gal80; LexAop‐CD8::GFP, LexAop‐hid*] only few remnants of the wrapping glia are detected (asterisks). In few A8 nerves some GFP localization is detected in the distal most parts (white arrow). (D) Control larva expressing GFP in wrapping glia with the genotype [*nrv2‐Gal4, 90C03‐Gal80; UAS‐CD8::GFP*]. Note all wrapping glia cover the segmental nerves. (E, F) Upon ablation of the wrapping glia in animals with the genotype [*nrv2‐Gal4, 90C03‐Gal80; UAS‐CD8::GFP, UAS‐hid*] slightly more GFP (white arrow) is detected along the nerves as compared to larvae with *LexA*‐mediated *hid* expression. (G, H) Exemplary cross section of nerves with remnants of wrapping glia (indicated by false color shading). The genotypes are indicated. (I) Number of nerve cross sections showing no wrapping glia debris. Single dots represent the percentage of nerves in one animal lacking any detectable wrapping glia processes.

**Figure S6.** Quantification of axonal area. (A) Box plot of axonal area for control and wrapping glia ablated larvae of different genotypes as indicated by the color code. WG indicates the wrapping glia driver [*90C03‐Gal80; nrv2‐LexA*]. The axonal area of cholinergic as well as glutamatergic axons increases upon wrapping glia ablation. (B) Box plots showing that the majority of axons in individual nerves is similarly affected by wrapping glia ablation. For details on statistical analysis see above.

**Figure S7.** Assignment of nerve identity exemplary shown for one third instar larval filet preparation. (A) Overview of a cross section through an entire larval filet. The genotype is indicated. WG indicates the wrapping glia driver [*90C03‐Gal80; nrv2‐LexA*]. Cholinergic axons were stained in the background of wrapping glia ablation. (B) Abdominal nerves A7–A3 of the left body side. (C) Abdominal nerves A7–A3 of the right body side. Scale bars are as indicated.

**Figure S8.** Assignment of nerve identity exemplary shown for one third instar larval filet preparation. (A) Overview of a cross section through an entire larval filet. The genotype is indicated. WG indicates the wrapping glia driver [*90C03‐Gal80; nrv2‐LexA*]. Glutamatergic axons were stained in the background of wrapping glia ablation. (B) Abdominal nerves A7–A3 of the left body side. (C) Abdominal nerves A7–A3 of the right body side. Scale bars are as indicated.

**Figure 9.** Nerve tracing across six segments is shown exemplarily for the left (A) and right (B) abdominal nerve A7, at the indicated position of segments A7–A2. The genotype is [*OK371‐Gal4/90C03‐Gal80; UAS‐APEX2* ^*m y r*^, *nrv2‐LexA/UAS‐APEX2* ^*myr*^, *LexAop‐hid*]. The number of cholinergic and glutamatergic axons is given below each image. Scale bars are as indicated.

**Figure S10.** Nerve tracing across five segments is shown exemplarily for the left (A) and right (B) abdominal nerve A6 at the indicated position of segments A6–A2. The genotype is [*OK371‐Gal4/90C03‐Gal80; UAS‐APEX2* ^*my r*^, *nrv2‐LexA/UAS‐APEX2* ^*my*r^, *LexAop‐hid*]. The number of cholinergic and glutamatergic axons is given below each image. Scale bars are as indicated.

**Figure 11.** Exemplary electron micrographs of APEX‐stained axons from cross sections of peripheral nerves. (A) *LexAop‐APEX2* ^*m y r*^ or (B) *LexAop‐Tau::APEX2* were driven by *ChAT‐LexA* while (C) *UAS‐mCD8::GFP::APEX2* was driven by ChAT‐Gal4. Note that no labeling difference can be detected. Scale bars are as indicated.

## Data Availability

The data that support the findings of this study are available from the corresponding author upon reasonable request.
