# Supplementary figures and images for: Cholinergic and Glutamatergic Axons Differentially Require Glial Support in the Drosophila PNS
Source: Glia. 2025 Mar 17;73(7):1365–82. doi: 10.1002/glia.70011 (PMC12121464; doi:10.1002/glia.70011)

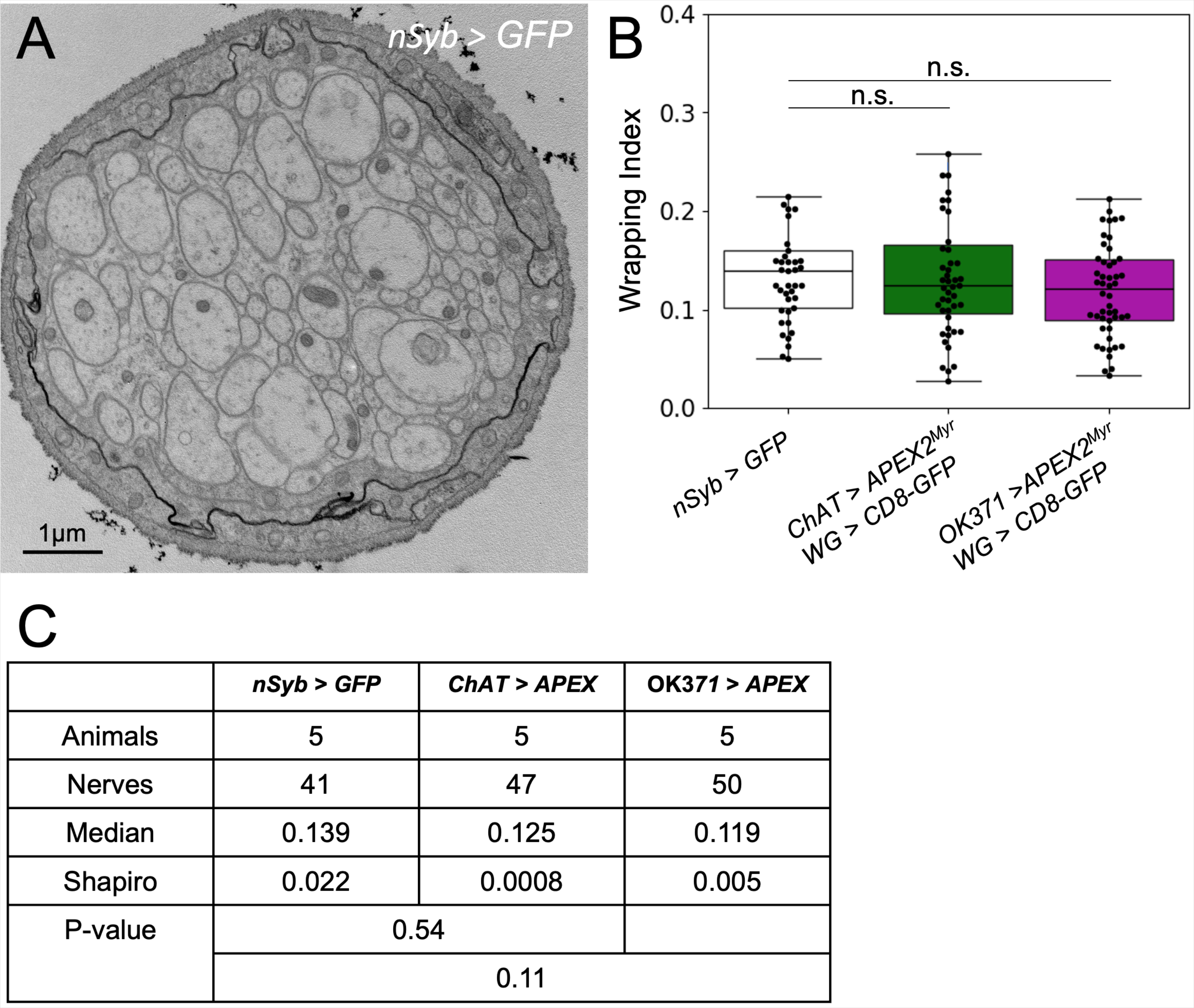

Supplement: Supplementary file 1 — Figure S1. Neuronal expression of APEX2myr does not affect axon wrapping. (A) Exemplary cross section of an abdominal nerve of an animal with the genotype [nsyb‐Gal4 UAS‐GFP]. Scale bar is 1 μm. (B) Box plot showing the distribution of wrapping indices of individual nerves from control larvae [nsyb‐Gal4 UAS‐GFP] and larvae expressing APEX2myr either in cholinergic [ChAT‐Gal4 UAS‐APEX2 myr ] or in glutamatergic neurons [OK371‐Gal4 UAS‐APEX2 myr ]. WG indicates the wrapping glia driver [90C03‐Gal80; nrv2‐LexA]. The median is indicated. (C) Statistics to (B). The numbers of analyzed animals and nerves is indicated. The Shapiro test was used to test for normal distribution of the data. Upon normal distribution a t test was used, otherwise a Mann–Whitney U test (MW test) was performed. Bottom and top of boxplots represent 25% and 75% percentile, respectively. Whiskers represent lower and upper quartile. [file GLIA-73-1365-s007.tiff]

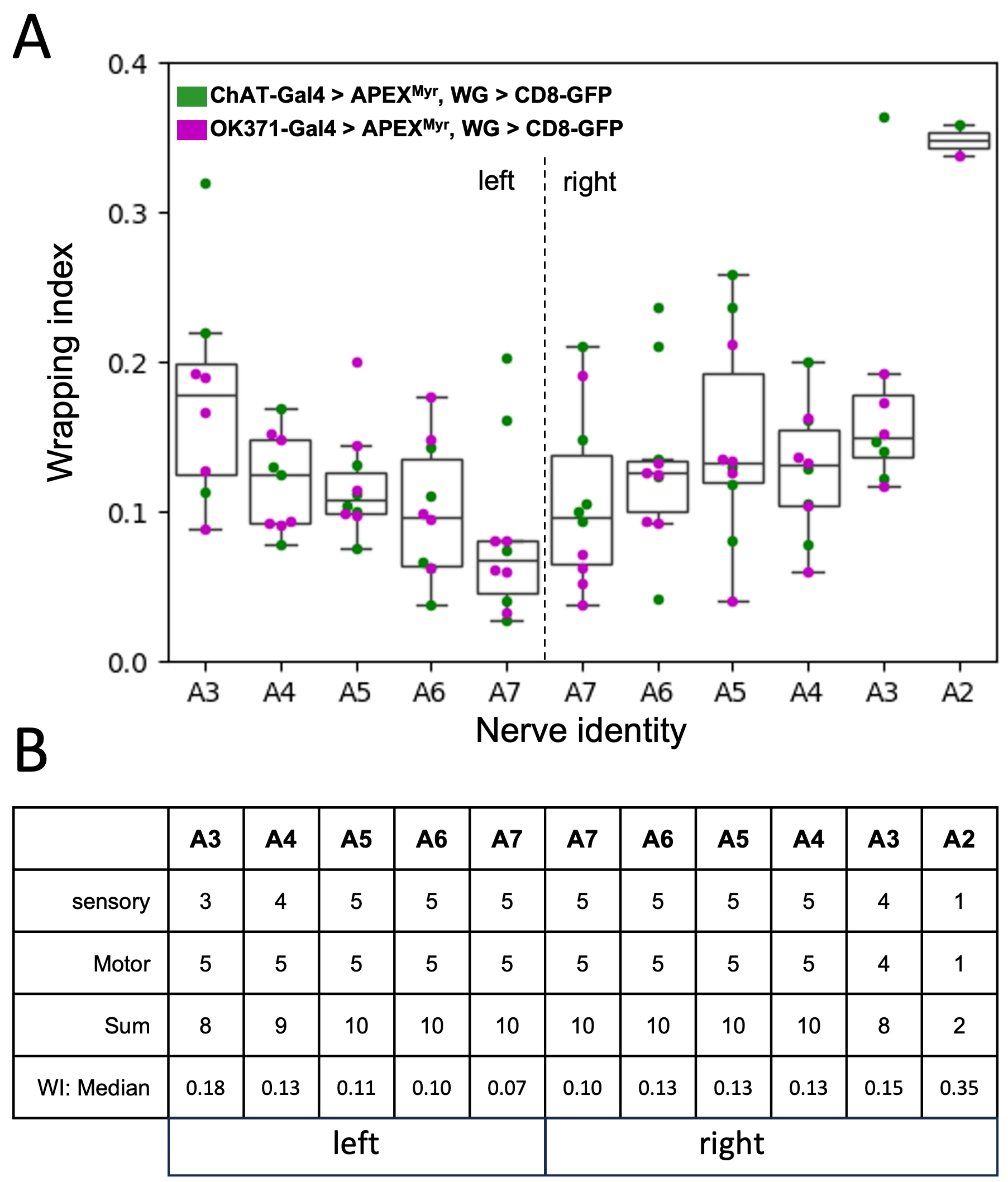

Supplement: Supplementary file 2 — Figure S2. The wrapping index at 150 μm distance from the ventral nerve cord correlates with the nerve identity. (A) Wrapping index of individual left and right abdominal nerves determined for animals that had labeled cholinergic axons (green dots) or glutamatergic axons (magenta dots). The abdominal nerves A3 and A4 are sectioned further distant to the exit point at the ventral nerve cord. Only the A8 nerve originates 150 μm away from the section plane. WG indicates the wrapping glia driver [90C03‐Gal80; nrv2‐LexA]. (B) Statistics to (A). The median is calculated based on all data points. [file GLIA-73-1365-s002.tiff]

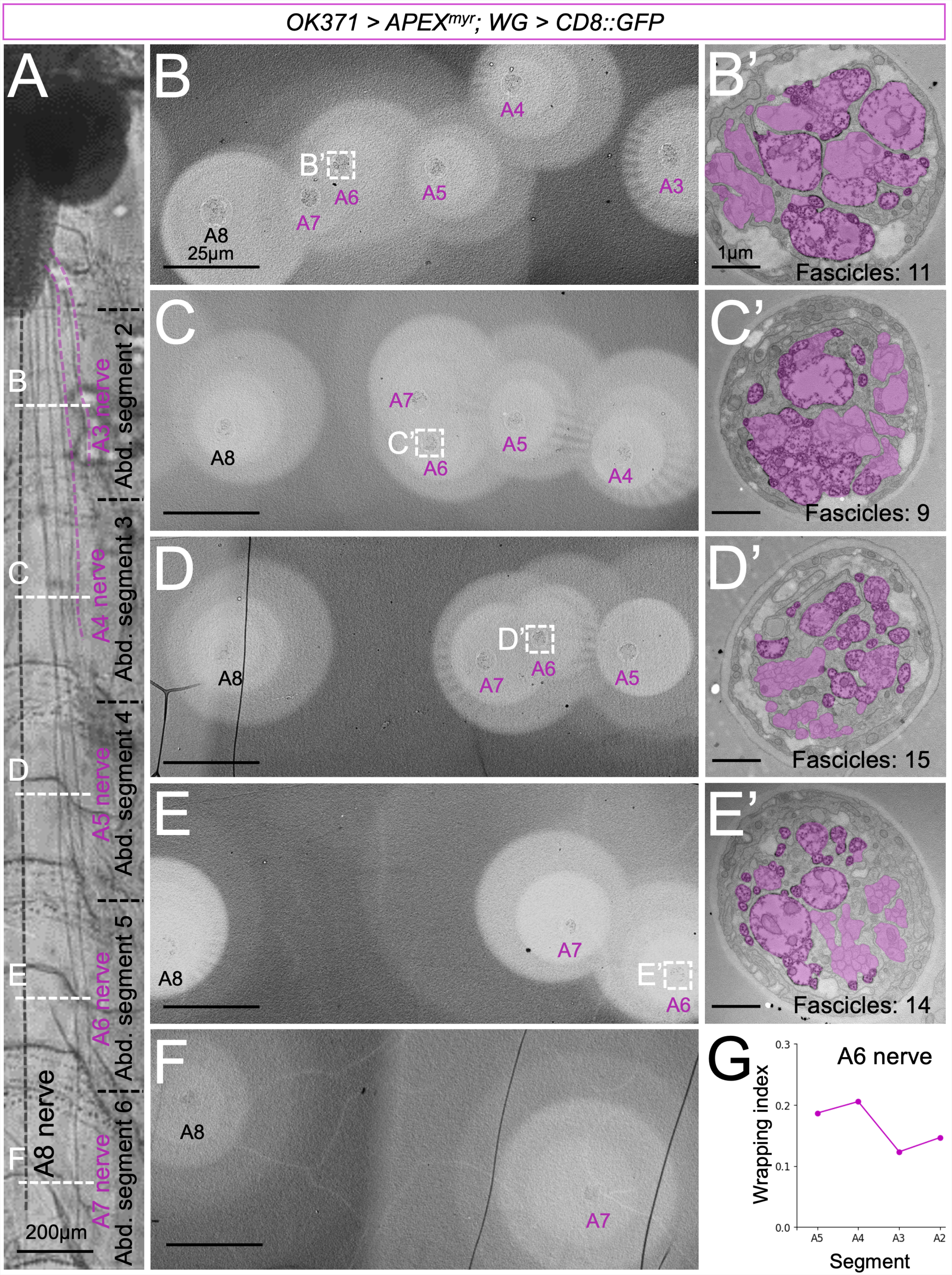

Supplement: Supplementary file 3 — Figure S3. The wrapping index changes over nerve length. WG indicates the wrapping glia driver [90C03‐Gal80; nrv2‐LexA]. (A) Light microscopic image of resin embedded, osmium stained third instar larval filet of the indicated genotype used for subsequent sectioning (B–F). (B–F) Overview electron microscopic images of sections taken at the positions indicated in (A). (B′–E′) High magnification images of the left A6 nerve showing the number of distinct fascicles indicated in false color (magenta). Note, that the glutamatergic motor axons are stained following APEX2myr expression and cluster to one side of the nerve. (G) The wrapping index of the left abdominal nerve A6 changes with sectioning position (see Figure S4 for remaining nerves). [file GLIA-73-1365-s003.tiff]

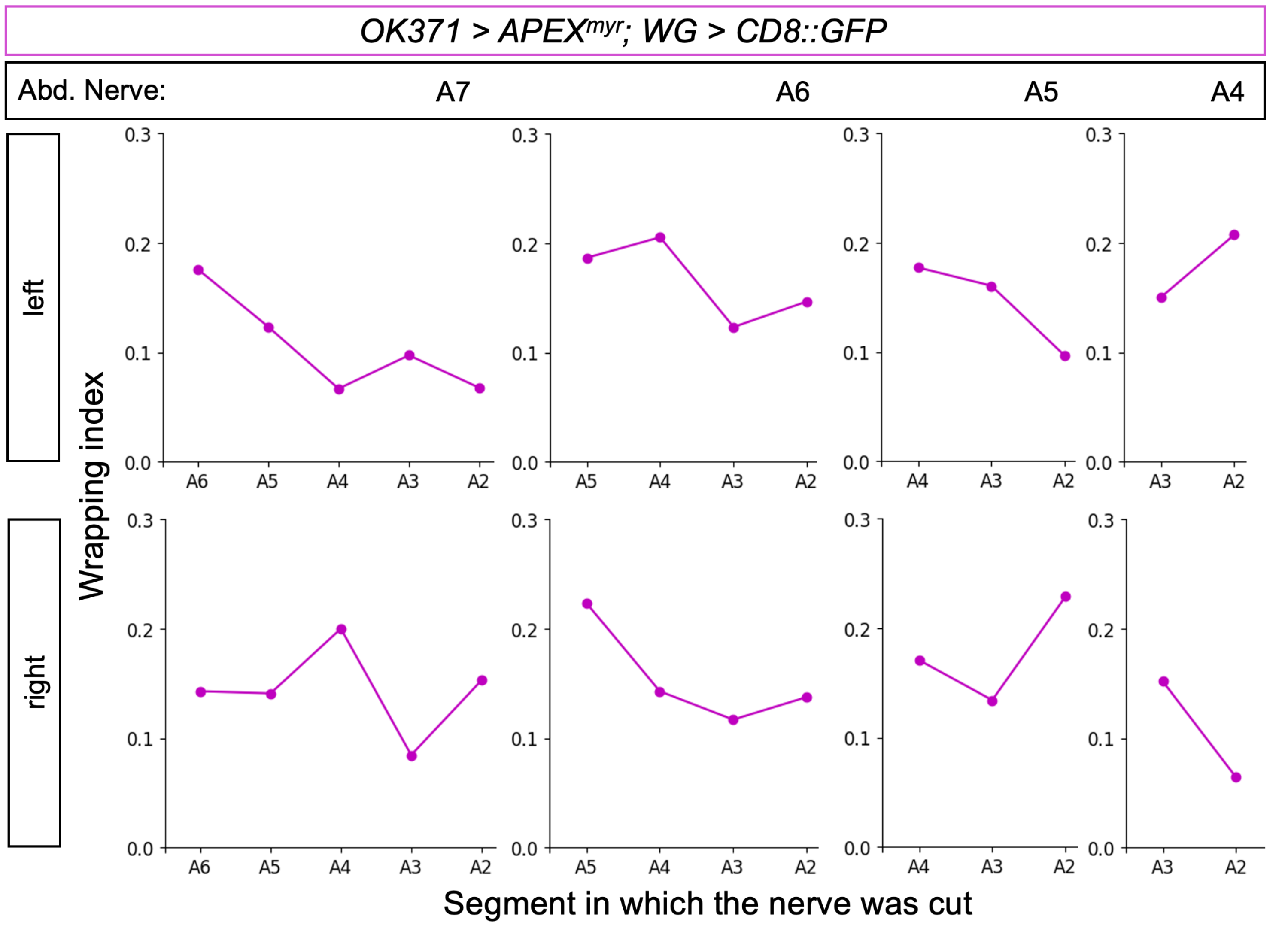

Supplement: Supplementary file 4 — Figure S4. The wrapping index changes over nerve length. The data are obtained from the same animal as in Figure S3. The wrapping index of abdominal nerves A4–A7 is plotted separately over the segmental position. [file GLIA-73-1365-s008.tiff]

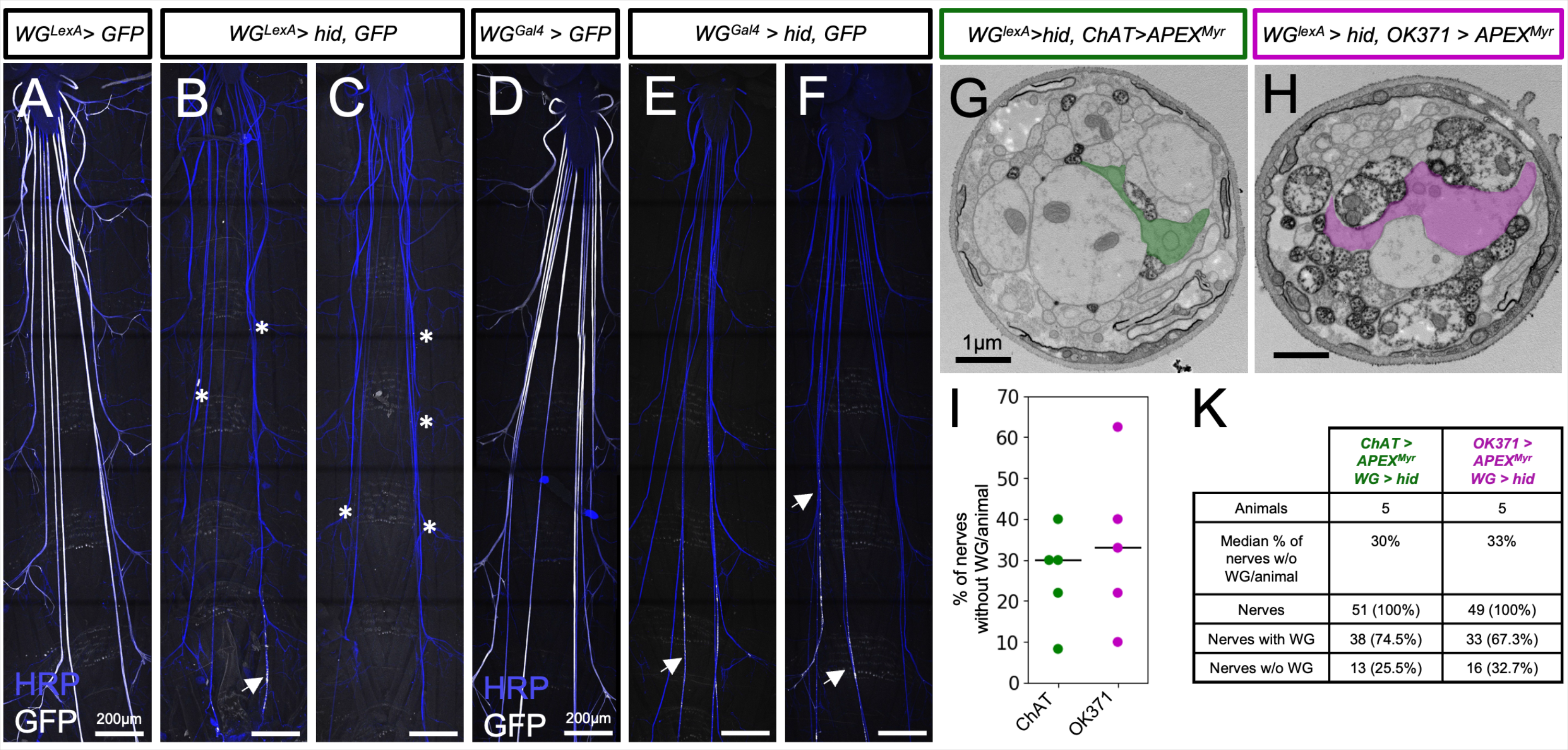

Supplement: Supplementary file 5 — Figure S5. Ablation efficacy. (A–F) Comparison of LexA and Gal4 mediated wrapping glia ablation (WGLexA or WGGal4). All neuronal membranes are stained using anti‐HRP antibodies (blue), GFP expression is shown in white. (A) Control larva expressing GFP in wrapping glia with the genotype [nrv2‐LexA, 90C03‐Gal80; LexAop‐CD8::GFP]. Note all wrapping glia cover the segmental nerves. (B, C) Upon ablation of the wrapping glia in animals with the genotype [nrv2‐LexA, 90C03‐Gal80; LexAop‐CD8::GFP, LexAop‐hid] only few remnants of the wrapping glia are detected (asterisks). In few A8 nerves some GFP localization is detected in the distal most parts (white arrow). (D) Control larva expressing GFP in wrapping glia with the genotype [nrv2‐Gal4, 90C03‐Gal80; UAS‐CD8::GFP]. Note all wrapping glia cover the segmental nerves. (E, F) Upon ablation of the wrapping glia in animals with the genotype [nrv2‐Gal4, 90C03‐Gal80; UAS‐CD8::GFP, UAS‐hid] slightly more GFP (white arrow) is detected along the nerves as compared to larvae with LexA‐mediated hid expression. (G, H) Exemplary cross section of nerves with remnants of wrapping glia (indicated by false color shading). The genotypes are indicated. (I) Number of nerve cross sections showing no wrapping glia debris. Single dots represent the percentage of nerves in one animal lacking any detectable wrapping glia processes. [file GLIA-73-1365-s006.tiff]

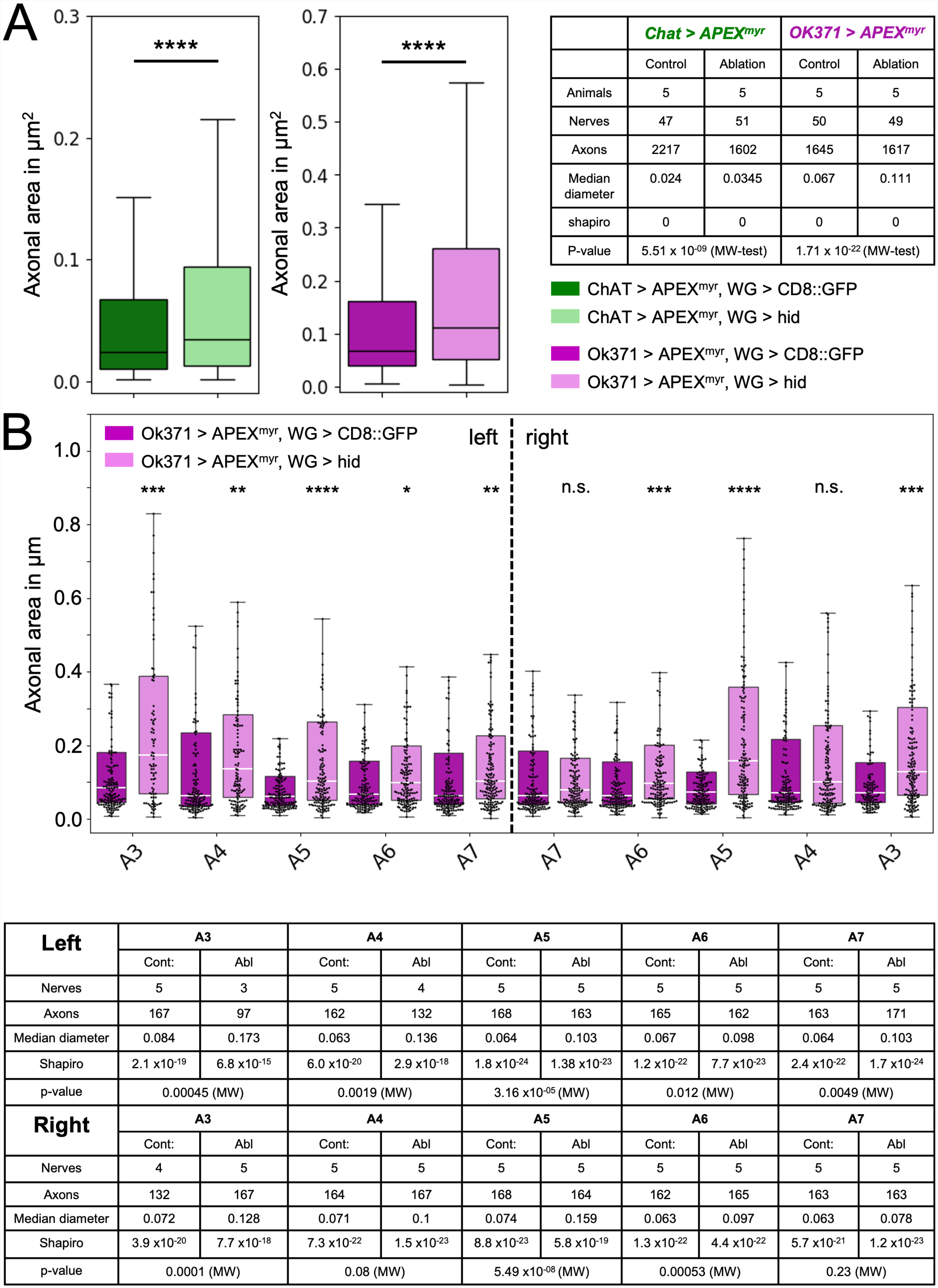

Supplement: Supplementary file 6 — Figure S6. Quantification of axonal area. (A) Box plot of axonal area for control and wrapping glia ablated larvae of different genotypes as indicated by the color code. WG indicates the wrapping glia driver [90C03‐Gal80; nrv2‐LexA]. The axonal area of cholinergic as well as glutamatergic axons increases upon wrapping glia ablation. (B) Box plots showing that the majority of axons in individual nerves is similarly affected by wrapping glia ablation. For details on statistical analysis see above. [file GLIA-73-1365-s001.tiff]

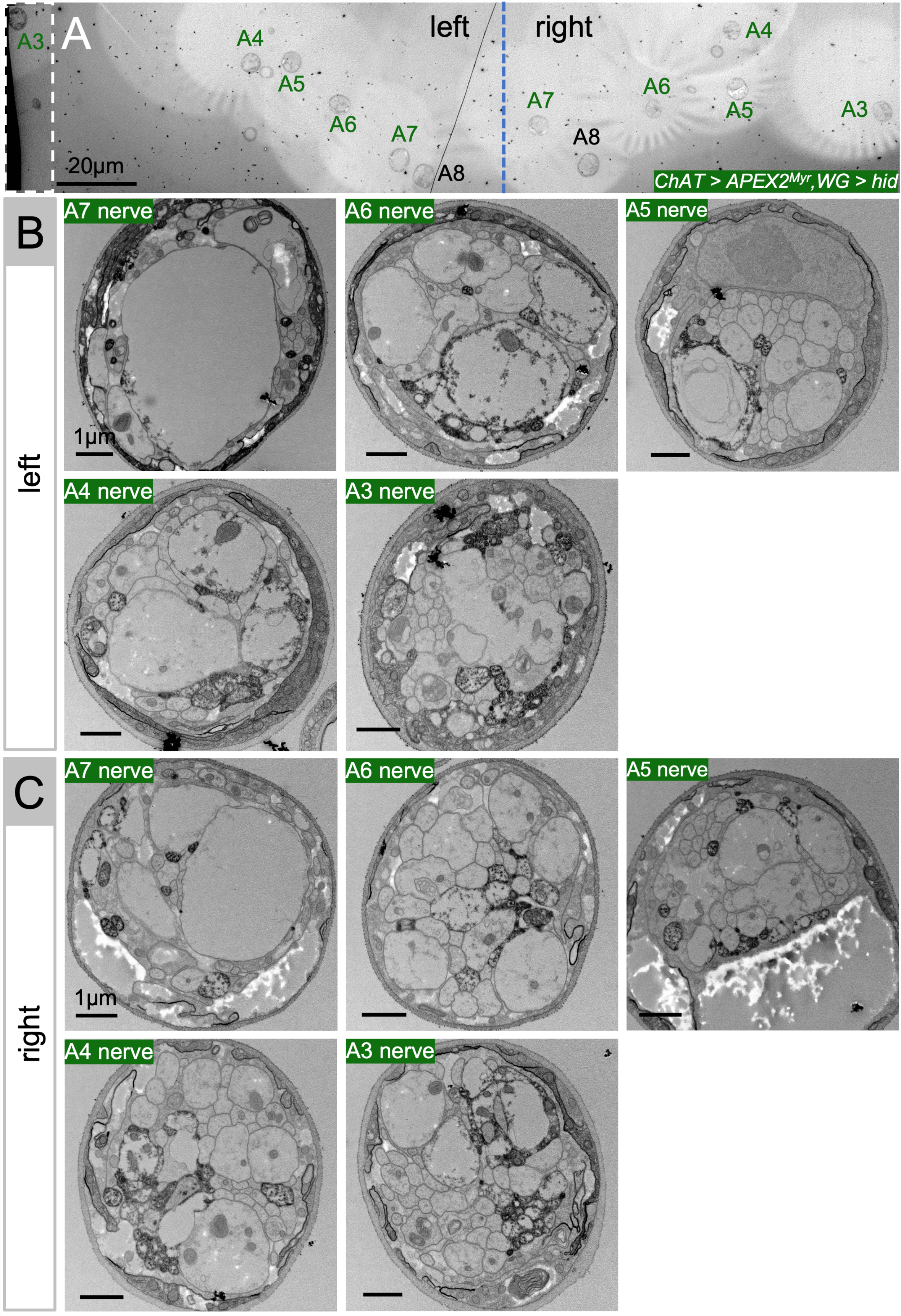

Supplement: Supplementary file 7 — Figure S7. Assignment of nerve identity exemplary shown for one third instar larval filet preparation. (A) Overview of a cross section through an entire larval filet. The genotype is indicated. WG indicates the wrapping glia driver [90C03‐Gal80; nrv2‐LexA]. Cholinergic axons were stained in the background of wrapping glia ablation. (B) Abdominal nerves A7–A3 of the left body side. (C) Abdominal nerves A7–A3 of the right body side. Scale bars are as indicated. [file GLIA-73-1365-s011.tiff]

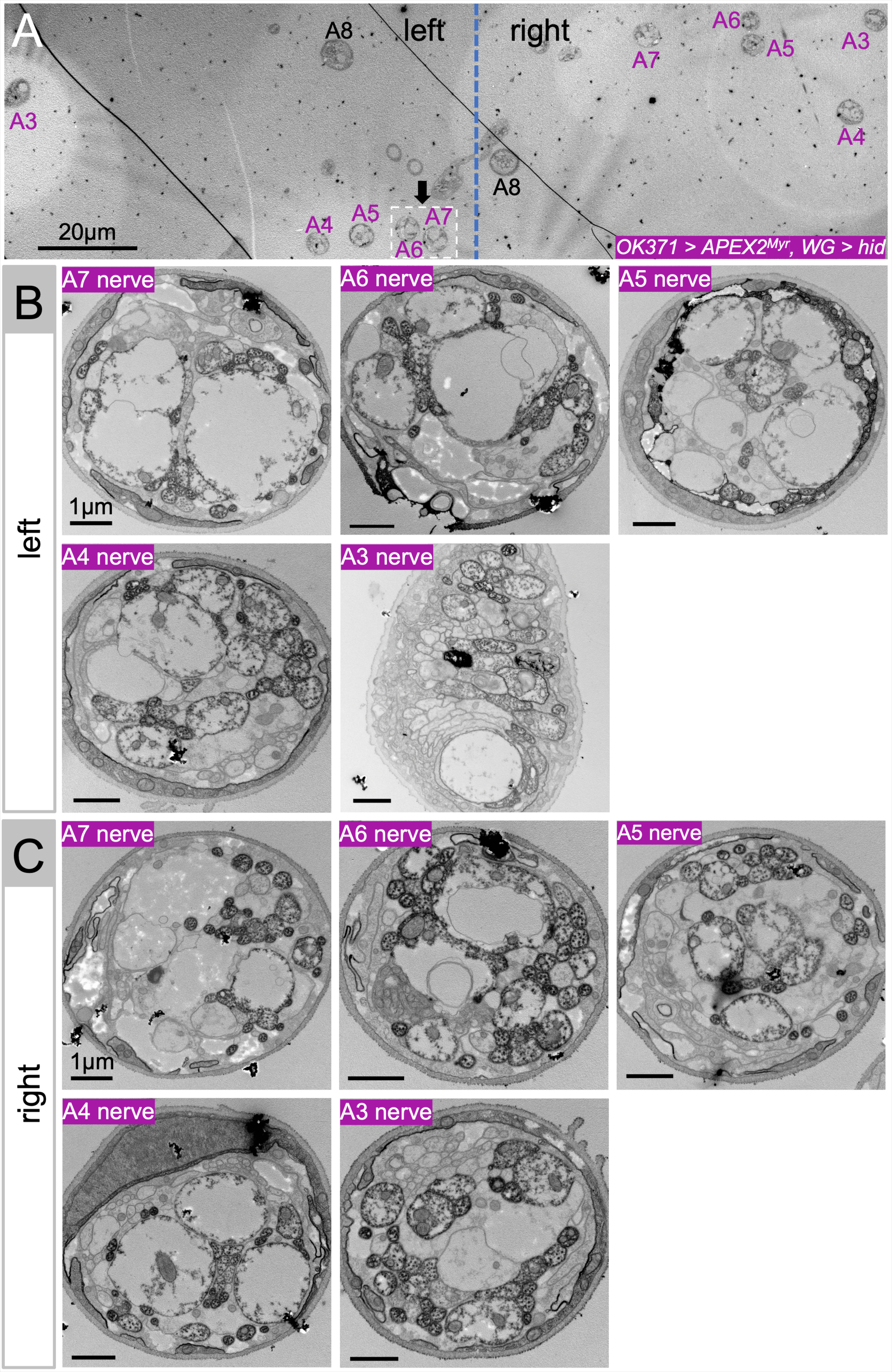

Supplement: Supplementary file 8 — Figure S8. Assignment of nerve identity exemplary shown for one third instar larval filet preparation. (A) Overview of a cross section through an entire larval filet. The genotype is indicated. WG indicates the wrapping glia driver [90C03‐Gal80; nrv2‐LexA]. Glutamatergic axons were stained in the background of wrapping glia ablation. (B) Abdominal nerves A7–A3 of the left body side. (C) Abdominal nerves A7–A3 of the right body side. Scale bars are as indicated. [file GLIA-73-1365-s004.tiff]

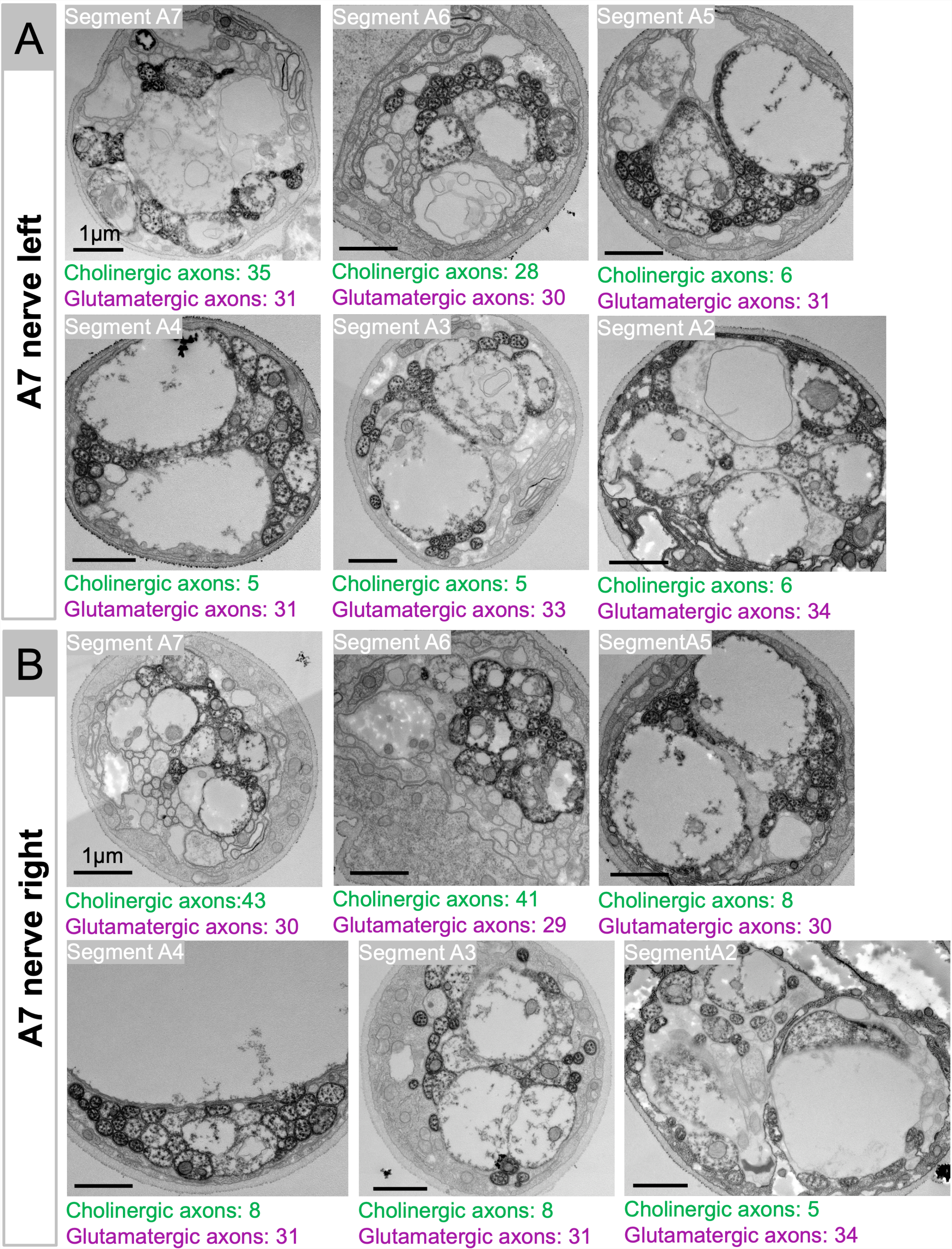

Supplement: Supplementary file 9 — Figure 9. Nerve tracing across six segments is shown exemplarily for the left (A) and right (B) abdominal nerve A7, at the indicated position of segments A7–A2. The genotype is [OK371‐Gal4/90C03‐Gal80; UAS‐APEX2 m y r , nrv2‐LexA/UAS‐APEX2 myr , LexAop‐hid]. The number of cholinergic and glutamatergic axons is given below each image. Scale bars are as indicated. [file GLIA-73-1365-s005.tiff]

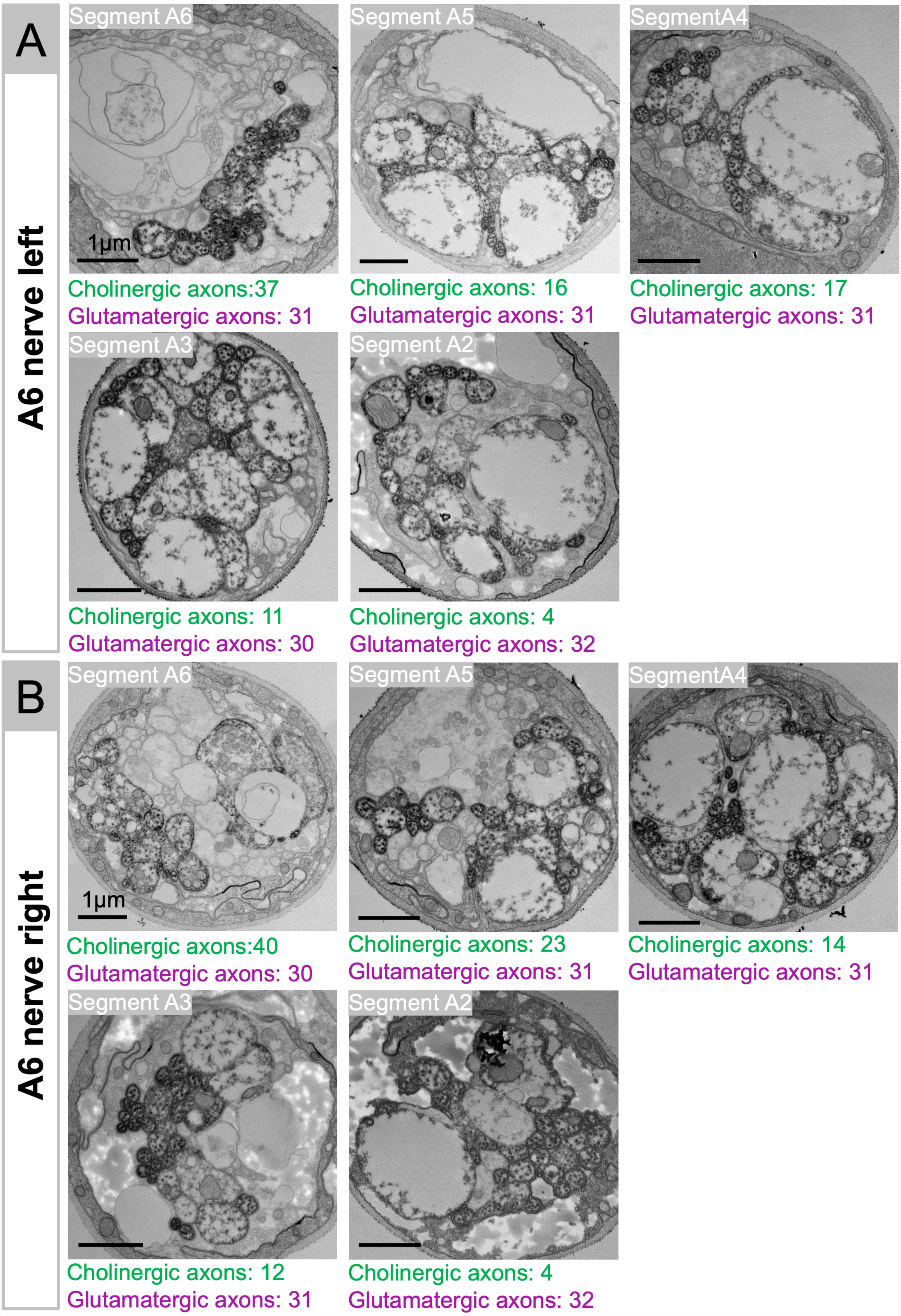

Supplement: Supplementary file 10 — Figure S10. Nerve tracing across five segments is shown exemplarily for the left (A) and right (B) abdominal nerve A6 at the indicated position of segments A6–A2. The genotype is [OK371‐Gal4/90C03‐Gal80; UAS‐APEX2 my r , nrv2‐LexA/UAS‐APEX2 myr, LexAop‐hid]. The number of cholinergic and glutamatergic axons is given below each image. Scale bars are as indicated. [file GLIA-73-1365-s010.tiff]

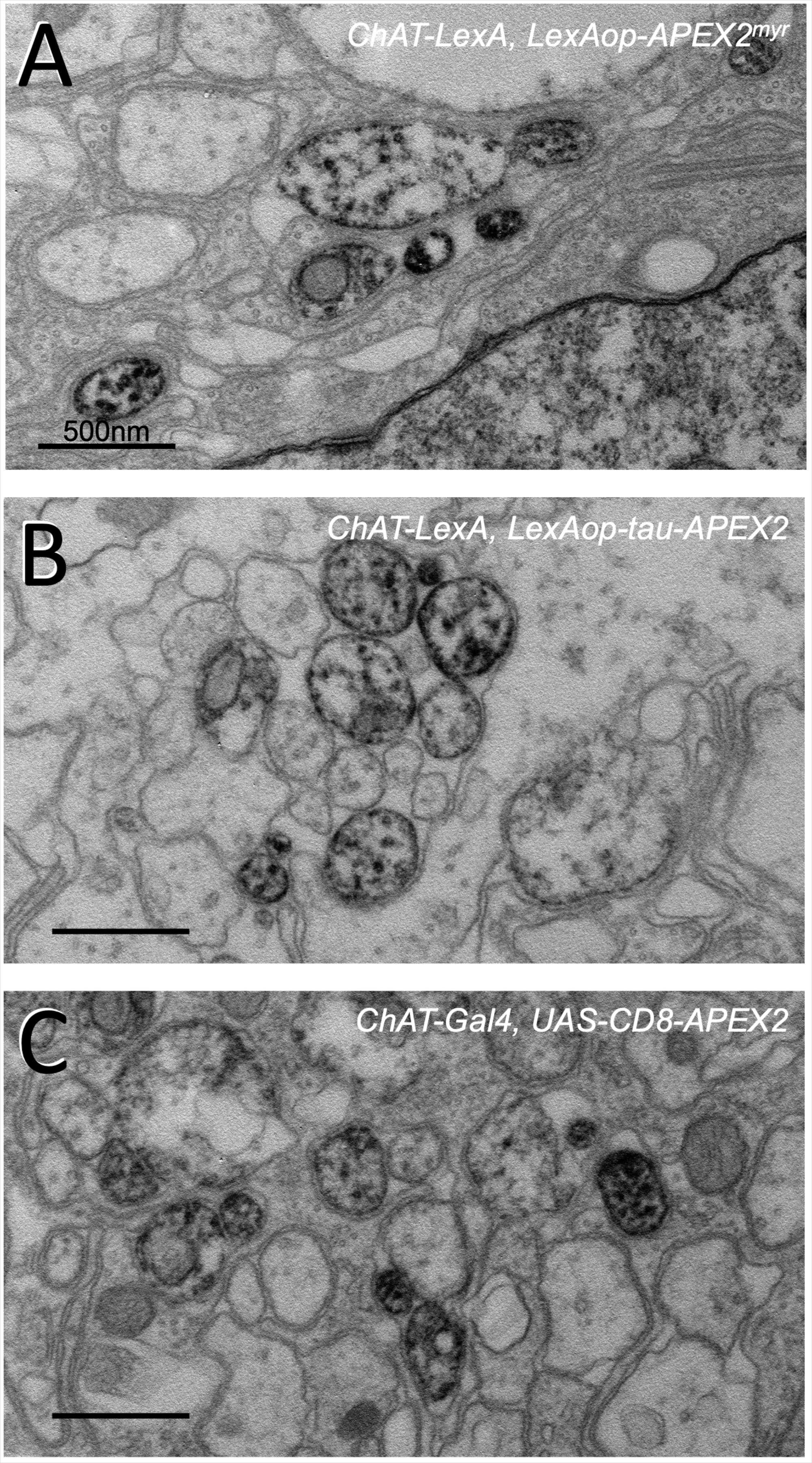

Supplement: Supplementary file 11 — Figure 11. Exemplary electron micrographs of APEX‐stained axons from cross sections of peripheral nerves. (A) LexAop‐APEX2 m y r or (B) LexAop‐Tau::APEX2 were driven by ChAT‐LexA while (C) UAS‐mCD8::GFP::APEX2 was driven by ChAT‐Gal4. Note that no labeling difference can be detected. Scale bars are as indicated. [file GLIA-73-1365-s009.tiff]
